# Transcriptomics of Differential Ripening in ‘d’Anjou’ Pear (*Pyrus communis* L.)

**DOI:** 10.3389/fpls.2021.609684

**Published:** 2021-06-16

**Authors:** Loren Honaas, Heidi Hargarten, John Hadish, Stephen P. Ficklin, Sara Serra, Stefano Musacchi, Eric Wafula, James Mattheis, Claude W. dePamphilis, David Rudell

**Affiliations:** ^1^USDA, ARS, Tree Fruit Research Laboratory, Wenatchee, WA, United States; ^2^Molecular Plant Sciences, Washington State University, Pullman, WA, United States; ^3^Department of Horticulture, Washington State University, Pullman, WA, United States; ^4^Tree Fruit Research and Extension Center, Washington State University, Wenatchee, WA, United States; ^5^Department of Biology, The Huck Institutes of the Life Sciences, Pennsylvania State University, University Park, PA, United States

**Keywords:** European pear, RNA-seq, ripening (fruit), biomarker, *Pyrus communis* (L.), fruit quality, random forest, co-expression network analysis

## Abstract

Estimating maturity in pome fruits is a critical task that directs virtually all postharvest supply chain decisions. This is especially important for European pear (*Pyrus communis)* cultivars because losses due to spoilage and senescence must be minimized while ensuring proper ripening capacity is achieved (in part by satisfying a fruit chilling requirement). Reliable methods are lacking for accurate estimation of pear fruit maturity, and because ripening is maturity dependent it makes predicting ripening capacity a challenge. In this study of the European pear cultivar ‘d’Anjou’, we sorted fruit at harvest based upon on-tree fruit position to build contrasts of maturity. Our sorting scheme showed clear contrasts of maturity between canopy positions, yet there was substantial overlap in the distribution of values for the index of absorbance difference (*I*_*AD*_), a non-destructive spectroscopic measurement that has been used as a proxy for pome fruit maturity. This presented an opportunity to explore a contrast of maturity that was more subtle than *I*_*AD*_ could differentiate, and thus guided our subsequent transcriptome analysis of tissue samples taken at harvest and during storage. Using a novel approach that tests for condition-specific differences of co-expressed genes, we discovered genes with a phased character that mirrored our sorting scheme. The expression patterns of these genes are associated with fruit quality and ripening differences across the experiment. Functional profiles of these co-expressed genes are concordant with previous findings, and also offer new clues, and thus hypotheses, about genes involved in pear fruit quality, maturity, and ripening. This work may lead to new tools for enhanced postharvest management based on activity of gene co-expression modules, rather than individual genes. Further, our results indicate that modules may have utility within specific windows of time during postharvest management of ‘d’Anjou’ pear.

## Introduction

Pome fruit ripening is a coordinated process that includes peel color change, softening, and acid loss. Pome fruit maturity advances during the growing season until fruit are picked. Ripening can occur on the tree, and if fruit are sufficiently mature at harvest, ripening can occur during the postharvest period. If not, fruit will lack the capacity to ripen meaning fruit will likely never meet consumer expectations. Fruit quality and ripening of European pear (*Pyrus communis*) is very complex and strongly dependent on various aspects of the orchard environment. This includes fruit position in the canopy and tree architecture (Layne and Quamme, 1975; Faragher and Brohier, 1984; Palmer et al., 1997; Fideghelli, 2007; Serra et al., 2016; Wu et al., 2018). The resulting variable light within the canopy has been linked to effects on fruit quality (Lakso, 1980; Ramos et al., 1994; Warrington et al., 1996; Awad et al., 2001; Stephan et al., 2008; Zhang et al., 2016). Indeed, large canopy trees (that have a more variable in-canopy environment) produce fruit crops with greater variability in fruit quality compared to planar-canopy trees (Musacchi, 2008; Zhang et al., 2016; Serra et al., 2018). Further, harvest time is a critically important factor when targeting the final fruit quality necessary to satisfy consumer expectations (Predieri et al., 2005; Vanoli and Buccheri, 2012; Blanckenberg et al., 2016). This all-together can lead to heterogeneous ripening among lots of fruit (Jajo et al., 2014), due in large part to variability in fruit maturity.

Given that harvest maturity is such a critical factor in pear fruit quality, a method to rapidly and non-destructively assess fruit maturity has been developed that utilizes near infrared/visible (NIR/vis) spectroscopy to calculate the index of absorbance difference [*I*_*AD*_, Ziosi et al. (2008)]. The *I*_*AD*_ provides a measure of chlorophyll-a content in the fruit based upon the difference between the absorbance of the chlorophyll-a peak at 670 nm and the spectrum background at 720 nm (Ziosi et al., 2008). It has been shown that there is a strong correlation between the *I*_*AD*_ estimate of chlorophyll-a content in fruit mesocarp and ethylene production during ripening, as well as a relationship between chlorophyll-a degradation and upregulation of ripening related genes including ACS1, ACO1, and polygalacturonase (PG) genes (Ziosi et al., 2008). This index, which reports lower values for more mature fruit, can be provided by a portable device called the differential absorbance (DA)-meter [Sinteleia, Bologna, Italy; Noferini et al. (2006)], thereby providing a rapid, non-destructive proxy for maturity. The *I*_*AD*_ index has been used to estimate maturity in horticultural studies that relate maturity and fruit quality in apples (Costamagna et al., 2013; Serra et al., 2016) and pears (Gagliardi et al., 2014; Jajo et al., 2014; Vidoni et al., 2015). Furthermore, as demonstrated by DeLong et al. (2014), the *I*_*AD*_ index can also be useful to predict risk for maturity-linked postharvest physiological disorders.

The primary component of *P. communis* postharvest management is cold storage at −0.5°C (Chen, 2016). Low temperature slows fruit ripening and delays or prevents physiological disorders and subsequent decay. For several *P. communis* cultivars, including ‘d’Anjou’, a period (often for weeks) at low temperature after harvest is required to initiate fruit ripening (Blankenship and Richardson, 1985). Additionally, extended cold storage in a controlled atmosphere (CA) where pO_2_ and pCO_2_ are low and high, respectively, relative to air, reduces ethylene action and further delays ripening compared to cold stored fruit held in air (Chen and Varga, 1997). The ethylene action inhibitor 1-methylcyclopropene (1-MCP) also delays ‘d’Anjou’ fruit ripening (Argenta et al., 2003), and the combination of 1-MCP treatment and cold storage in CA provides the longest ripening delay. But this combination also causes uncertainty as to when (or if) ripening capacity is regained (Calvo, 2002; Wang, 2016); the subsequent ethylene treatment of 1-MCP-treated pears does not promote ripening capacity (Wang et al., 2015).

Therefore, current postharvest technology provides sufficient capability to *slow* pear fruit ripening and senescence, yet an effective combination of postharvest management tools to produce fruit that ripen *predictably*, or tools to predict ripening capacity, are lacking (Wang et al., 2015). This is due in part to a lack of knowledge about the molecular machinery that controls fruit maturity and ripening (Lu et al., 2018). New postharvest tools, based on biosignatures (one or more biomarkers, e.g., metabolic and/or gene activity profiles with predictive or diagnostic value, deployed at various time points), could be developed as we learn about the molecular processes underway as the capacity to ripen is initiated, as well as those that drive the ripening processes.

In this study we sought to fill knowledge gaps in molecular processes that influence maturity and to begin the development of tools for prediction of fruit ripening capacity. To do this, we created contrasts of maturity by harvesting fruit from different canopy positions. We then sorted fruit into classes based on *I*_*AD*_ values. This allowed us to explore contrasts of maturity (i.e., within classes) that were too subtle for differentiation by *I*_*AD*_ value. We also used the results of the fruit quality analysis to guide our transcriptome analysis. We aimed to identify gene expression-based biosignatures that could help us understand the different ripening characteristics of the fruit in a narrow classification–that is, fruit from different canopy positions that differed in their maturity and ripening characteristics, but could not be differentiated by *I*_*AD*_. Against the backdrop of massive shifts in gene expression (>18,000 differentially expressed genes – DEGs) through 8 months of storage, we found smaller numbers of DEGs at each time point between fruit harvested from different canopy positions. Even though the number of DEGs was roughly similar at each time point, the gene expression profiles were distinctive, indicating successive changes of gene expression driven by at-harvest differences. Because the fruit quality analysis revealed differences of fruit maturity in our narrow classification, we searched for gene activity with a phased character that reflected the phased at-harvest fruit maturity. Using machine learning and a novel approach of condition-specific co-expression, we identified hundreds of genes with such a phased character, providing the basis for a better understanding of genes involved in the control of ripening. These genes help build the foundation for a postharvest molecular toolkit by providing a putative biomarker suite that might be useful to estimate ripening capacity of ‘d’Anjou’ pear at various points during storage.

## Materials and Methods

### Experimental Site, Plant Material Selection, and Fruit Harvest

We selected a commercial orchard in Cashmere, WA (United States, 47° 31′ 22.3″ N, 120° 30′ 41.1″ W) considered as representative of the low-density pear cultivation system still widely adopted in the Pacific Northwest region of North America. Trees (‘d’Anjou’ pear grafted on ‘Bartlett’ seedling rootstock) had open vase architecture (∼4 m high), a planting distance of 6 m × 6 m, and were oriented east to west. Standard horticultural practices were adopted in terms of pest and disease control; irrigation was provided by micro-sprinklers. Trees in this block were characterized by (1) large canopies with heterogeneous production with more fruit in medium-high layers of the canopy, (2) a wide range of light interception in the different areas of the canopy, (3) variability in fruit development, and (4) significant variability of fruit quality at harvest and during the postharvest period (Serra et al., 2018).

Before harvest, trees (*n* = 15) were mapped by the use of a portable light measuring system made of a light-bar (Accu-PAR LP-80, Decagon Devices, Pullman, WA, United States), a data acquisition circuit and data acquisition card (NI 9205, National Instruments, Austin, TX, United States) connected to a laptop (Dell Latitude E6430 ATG) to define canopy positions (Zhang et al., 2016). This Photosynthetically Active Radiation (PAR) system consisted of a light-bar with a total measuring length of 2.4 m, resulting in 24 readings (0.1 m/reading) as described in Zhang et al. (2016). The canopy positions were defined based on midday measurements with the light bar placed 2.0 m and 3.5 m off the ground [following Zhang et al. (2016)]. Approximating commercial practice, the harvest date was determined based on a firmness standard [58–67 N, (Bai et al., 2020)]. We picked and sorted pears based on predefined canopy positions (external canopy = 70–100% light interception, internal = <30% light interception). After harvest, pears were placed in cold air storage (4°C) until further evaluation and sorting.

### Fruit Sorting and Storage

Inside the cold room, fruit weight (g) and *I*_*AD*_ (Ziosi et al., 2008) was assessed on 1,013 pears picked from external locations in the tree canopy and 934 pears from internal locations by the use of an infra-red (IR) sensor scale (Ohaus NavigatorXT NVT1601/1, Ohaus Corporation, Parsippany, NJ, United States) and the DA meter (TR-Turoni, Forlì, Italy). Pears weighing below 180 g and above 300 g were discarded to create a commercially representative sample of fruit. At the time of assessment by the DA meter, the range of all pears was *I*_*AD*_ of 0.97 to 2.16.

**FIGURE 1:**
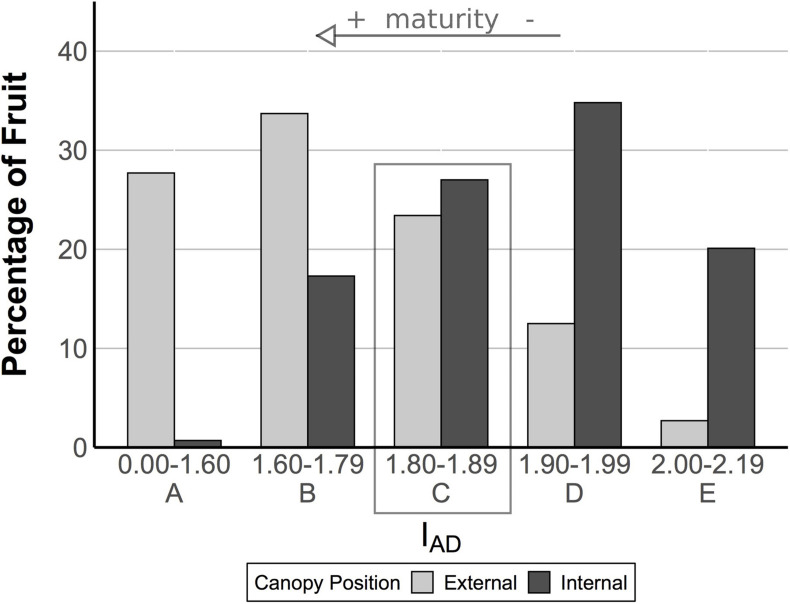
Fruit from the two canopy positions have an overlapping distribution of *I*_*AD*_. The *I*_*A*__*D*_ provides a measure of chlorophyll-a content in the fruit based upon the difference between the absorbance of the chlorophyll-a peak at 670 nm and the spectrum background at 720 nm. It has been used as a proxy for maturity in pome fruits (see arrow). In this experiment, we analyzed fruit quality by *I*_*AD*_ class. Within class C (see box), fruit were ostensibly of similar maturity, but our analysis of fruit quality revealed different postharvest outcomes, suggesting external fruit were more mature. We targeted class C fruit for a transcriptome analysis to search for genes associated with these different postharvest outcomes.

To further sort and classify fruit, an *I*_*AD*_ distribution was created using *I*_*AD*_ values from all internal and external fruit to create maturity classes, and from this distribution, fruit were then further segregated into approximate maturity classes based on their *I*_*AD*_ value (Figure 1). This resulted in an overlapping classification from class A (more ripe) to class E (less ripe) for all fruit (Figure 1). *I*_*AD*_ class C had the most similar proportion of internal and external pears (27.0 vs 23.4%, respectively). Therefore, ripeness in pears from class C is not readily distinguishable and these fruit were selected for transcriptome analysis to further examine differences between pears from different canopy positions with ostensibly similar at-harvest maturity. Fruit belonging to each maturity class were randomized and subdivided into four groups and sampled for fruit quality and RNA extraction (only for class C pears) at the following four time points: after harvest, T0 (no CA storage), immediately after removal from CA storage at three time points (3, 6, and 8 months – T1, T2, and T3, respectively). Long-term storage fruit were held in research CA room (−0.5°C, 2 kPa O_2_, 0.8 kPa CO_2_) in a commercial warehouse. Fruit quality was also assessed on stored fruit after a 7-day ripening period at 20°C.

### Fruit Selection and Tissue Sampling for RNA Extraction

Fruit peel and cortex tissues were sampled from cold pears; for the “harvest” time point this was done at 4°C after brief air storage, and for long-term fruit this was done at 0.5°C within 1 day after removal from CA storage. Fruit for tissue sampling was selected to have similar *I*_*AD*_ (after storage), similar *I*_*AD*_ drop following harvest (e.g., *I*_*AD*_ [T1] – *I*_*AD*_ [T0]), similar weight, and relative absence of surface defects, punctures and with intact stem where possible. Tissue from three individual pears was pooled to create a biological replicate. A total of five biological replicates were collected from *I*_*AD*_ class C (1.80–1.89 *I*_*AD*_), for a total of 80 samples used for RNA extractions (2 canopy positions × 2 tissue types × 4 time points × 5 biological replicates × 1 *I*_*AD*_ class). Prior to sampling, fruit were washed in deionized water and let air-dry for 1 min. Peel was sampled using a vegetable peeler from the entire fruit and the cortex samples were obtained by pooling together three wedges from the equatorial ring of each pear. Care was taken to minimize cortex tissue contamination of peel samples. Samples were flash frozen in liquid nitrogen, ground to fine powder with IKA^®^ A11 basic rotary mill (IKA^®^ Works, Inc. Wilmington, NC, United States) and stored at −80°C.

### Fruit Quality Analysis

At each time point, before removal from cold storage, characterization of the pears (15 fruits per canopy position) was performed and included fruit weight and *I*_*AD*_ index assessment prior to the tissue sampling for transcriptomic analysis. Soluble solids content (SSC) was measured (PAL-1, ATAGO, USA Inc., Bellevue, WA, United States) on the remaining portions of individual fruit after tissue sampling for transcriptome analysis. Titratable acidity and pH were assessed (TIM850 titrator, Radiometer, Lyon, France) at all postharvest time points on the composite fruit chunks representing each replication (20 values per each canopy position).

A larger set of fruit following the previously described at-harvest sorting protocol was dedicated to comprehensive fruit quality analysis. Additional color parameters included percentage of peel over-color surface (red blush), background color by CIELAB coordinates L^∗^ (lightness, 0 = black, 100 = white), a^∗^ (green–red), b^∗^(blue–yellow) (Minolta CR-300, Osaka, Japan), and according to McGuire (1992) and Nunez-Delicado et al. (2005), Chroma (C), Hue angle (h) were calculated. Fruit were also visually assessed for superficial scald incidence and other defects at 3, 6, and 8 months of CA storage. Fruit ethylene production was measured accordingly to Fan et al. (1998) and pear firmness was measured using a MDT-1 penetrometer with an 8 mm probe (Mohr and Associates, Richland, WA, United States) on both cheeks after a section of peel was removed and values were averaged for each pear. For each time point except for harvest, instrumental fruit quality was assessed following a period of at least 12 h of equilibration to room temperature after CA removal (“unripe” stage), as well as after 7 days (“ripe” stage) at 20°C in a ripening room.

### RNA Extraction, Quality Control, and Transcriptome Sequencing

Total RNA was extracted from cryopreserved tissue of the 80 biological samples (described above) as described in Honaas and Kahn (2017). RNA purity, quantity, and integrity was evaluated as described in Honaas et al. (2019). High quality total RNA (RIN ≥ 8.0) was used as input for Illumina TruSeq^1^ (RS-122-2103, Illumina San Diego, CA United States) library preparation at the Penn State Genomics Core facility (University Park, PA, United States) as described in Honaas et al. (2019). Libraries were sequenced on a 150 bp single-end protocol to a target volume of ∼20 million reads per biological replicate on Illumina’s HiSeq 2,500 in Rapid Mode. Read data are publicly available at the Sequencing Read Archive^2^ (BioProject ID PRJNA715928). The expression matrix is available in Supplementary File 1.

### Reference Selection, Transcriptome Data Processing and Read Mapping

The BartlettDH v2.0 genome (Linsmith et al., 2019) was ultimately selected as the reference for RNA-seq analysis, though initial validations (PCA, qPCR – see below) were performed using a gene expression matrix (GEM) created with the Bartlett v1.0 reference (Chagne et al., 2014) fetched from the Genome Database for Rosaceae [GDR^3^, Jung et al. (2019)]. The predicted transcripts of BartlettDH v2.0 (*Pyrus communis* Bartlett DH v2.transcripts.fasta.gz) were downloaded from GDR. Raw reads were first examined with FASTQC^4^ to determine the quality of reads and identify embedded and partial TruSeq3 adaptor sequences. Reads were then trimmed to remove adaptor sequences and low-quality regions using Trimmomatic (v0.36) (Bolger et al., 2014) as described in Honaas et al. (2019). Cleaned reads were mapped to the predicted transcripts of BartlettDH v2.0 and expression abundance estimated in a strand-specific mode using the RSEM (v1.3.0) pipeline (Li and Dewey, 2011) with the inbuilt Bowtie2 (Langmead and Salzberg, 2012) read aligner option to construct a GEM. The mean mapping rate of the ‘d’Anjou’ RNA-seq data to BartlettDH v2.0 (Linsmith et al., 2019) gene models (mRNAs) was 63.8%. This is an improvement from previous work by Wang et al. (2017) who reported 58.1% of ‘Red Bartlett’ and ‘Starkrimson’ pear transcriptome data mapped to Bartlett v1.0 (Chagne et al., 2014), as well as an improvement over our pilot read mapping analysis that showed a mapping rate of these data reported here to Bartlett v1.0 of 56.1%.

### Validation of RNA-Seq Data

A principal component analysis (PCA) was performed on normalized counts from peel tissues using NIPALS algorithm in Unscrambler X, (Camo, Trondheim, Norway). The model is presented using Origin Pro graphing software (Northampton, MA, United States). This analysis confirmed that the expression data had sufficient structure to distinguish fruit through time and between canopy positions (Supplementary Figure 1). RNA-seq estimates of gene activity were then validated on a subset of genes via qPCR. The subset of genes selected was based on the workflow described in Hargarten et al. (2018). The following criteria were used to select genes: (1) cumulative reads per kilobase transcript per million reads (RPKM) ≥ 2,000 (*n* = 1,830 genes), (2) standard deviation between 50–100 RPKMs (*n* = 47), (3) linear relationship with time in a subset of samples (*n* = 19), (4) high identity matches between *de novo* ‘d’Anjou’ transcripts and Bartlett genome reference. ‘d’Anjou’ pear transcripts [assembled *de novo* with CLC WorkBench V9.0^5^ as described in Honaas et al. (2016)] were searched with BLASTn using the coding sequence of the 19 Bartlett v1.0 candidates as queries. Nucleotide alignments of *de novo* ‘d’Anjou’ transcripts to Bartlett v1.0 reference sequences were performed using Geneious (v9.1.8). The 10 sequences with the highest overall similarity to Bartlett v1.0 reference sequences (Supplementary Table 1) were selected for qPCR validation. qPCR was performed as described in Hargarten et al. (2018) on the subset of RNA samples listed in Supplementary Table 2 using primers listed in Supplementary Table 3.

For comparisons between the GEMs made with Bartlett v1.0 and BartlettDH v2.0, the 10 validation gene sequences (transcripts from both Bartlett v1.0 and the ‘d’Anjou’ *de novo* assembly) were used to search BartlettDH v2.0 transcripts. Normalized qPCR expression and normalized RNA-seq counts (RPKMs) from BartlettDH v2.0 best matches for the 10 validation genes were regressed using Pearson’s r.

### Differential Expression and Functional Enrichment Analysis

Differential expression (DE) analysis was performed using R (R Core Team, 2019) and the package ‘DESeq2’ v1.28.1 (Love et al., 2014). Alpha (with Bonferroni correction) was set to 0.05. The pairwise tests for DE analysis for both peel and cortex tissue are listed in Supplementary Table 4. For functional enrichment analysis, the Gene Ontology (GO) annotation file for BartlettDH v2.0 was downloaded from GDR (PCommunis_DH_v2.0_genes2GO.xlsx.gz). The functional enrichment analysis was performed using the R package ‘topGO’ v2.36.0 (Alexa and Rahnenfuhrer, 2019). To identify enriched GO terms among genes of interest, we supplied the topGOdata object builder function with a list of the BartlettDH v2.0 gene names and their corresponding GO terms via the annFUN.gene2GO function. Using the topGOdata object, GO enrichment significance was determined using the getSigGroups function with the “*classicCount*” enrichment and “*weight01Count*” algorithm (taking GO hierarchy into account) to execute Fisher’s Exact tests for significance; alpha was set to 0.05.

### Visualization of Gene Expression With Heatmaps

To examine global expression patterns, normalized count data (RPKM) were clustered using agglomerative, hierarchical clustering. For hypothesis generation, agglomerative clustering can have superior performance over decomposition methods in smaller datasets, and when there is no *a priori* knowledge of cluster numbers (Saelens et al., 2018). The distance metric was calculated using Euclidean distance to account for non-linear and inverse relationships as well as outliers and skewed distributions (Priness et al., 2007; Yip and Horvath, 2007; Song et al., 2012; Saelens et al., 2018). To determine which clustering method best represented our data, we ran a Pearson’s correlation in base R (R Core Team, 2019) using the cor function between distance measurements determined by the dist function and the cophenetic distance (computed after clustering) using the cophenetic function on a subset of our data. Correlations were performed with average linkage (*R* = 0.99). To visualize clusters, heatmaps were generated using the R package ‘gplots’ v3.0.3 (Warnes et al., 2020) and the function heatmap.2. Z-score scale transformation of RPKM expression data was performed prior to clustering using the R package ‘massageR’ v0.7.2^6^ and the function heat.clust to emphasize patterns in the data for hypothesis generation (Altman and Krzywinski, 2017).

### Feature Selection via Machine Learning

Before additional RNA-seq analysis, feature selection was carried out using GSForge^7^ to select features (genes) that are most likely to underlie the variance that corresponds to the data labels (i.e., external and internal position or cortex and peel tissue), thereby reducing the number of genes used for downstream analyses. GSForge is a Python library that supports feature selection using the Boruta algorithm (Kursa et al., 2010). The Boruta algorithm avoids overfitting of the data model by comparing an initial set of selected features with a set of *n* randomized data sets that also undergo feature selection. Sample labels are randomly reordered for each set. Features whose scores are higher than the randomized sets are considered important. The RandomForestClassifier from the scikit-learn package^8^ was used and Boruta was instructed to perform 1,000 iterations. This generated a list of 830 genes which were used for downstream network analysis. A Jupyter Notebook was used to perform this analysis and is included in Supplementary File 2.

### Condition-Specific Gene Co-expression Network Construction

KINC v 3.4.2 (Shealy et al., 2019) was used for condition-specific gene co-expression network (csGCN) construction. KINC was used rather than other network construction tools because it reduces false edges by ensuring statistical assumptions are met at each pairwise comparison, removes biased correlations, and can identify co-expression between genes that have different and distinct condition-specific modes of expression. Such distinct modes may confound traditional correlation approaches. KINC uses Gaussian Mixture Models (GMMs) to cluster pairwise gene expression and identify clusters (or modes) of expression. These clusters then undergo correlation independently followed by association testing with sample labels (i.e., tissue or canopy position). A gene pair may therefore have multiple edges, with each associated to different labels. The result is a network where edges are assigned condition-specific *p*-values (i.e., for tissue type and canopy position). Furthermore, by assigning condition-specific *p*-values to edges, we can filter non-significant relationships to explore edges that may have lower correlation values, but are still perhaps meaningful, and which are often excluded by other network construction tools. Additionally, using *p*-values to extract condition-specific network subgraphs allows us to explore relationships between multi-functional genes while avoiding the “hairballs” that are present with most correlation networks and which are hard to interpret.

Here, the GEM for the 830 genes selected using Boruta provided input to create the csGCN. KINC was instructed, using the similarity function, to only consider GMM clusters with at least 15 samples. Next, clusters underwent a power analysis test (alpha: 0.001, power = 0.8), using the corrpower function, to ensure lowly powered clusters are removed. Remaining clusters were tested using two z-score tests of proportions (alpha: 0.001) for association with tissue, canopy position and combined tissue/canopy position; and a list of candidate network edges was exported in tidy format using the extract function. Lastly, the set of final edges was determined by testing and removing biased edges with the kinc-filter-bias.R script and edges were ranked by importance using the kinc-filter-rank.R script (both scripts are part of the KINC software). For steps just described, all parameters not specifically mentioned were set as default. Step-by-step Linux command-line instructions, including parameters are included in Supplementary File 2. Visualization of the resulting network was accomplished using both the built in KINC 3D viewer and Cytoscape (Smoot et al., 2011).

### Phased Edge Detection

KINC was able to identify large csGCNs associated with tissue type (cortex or peel), but found few edges associated with canopy position (external or internal) or the combined position and tissue (external cortex, external peel, internal cortex, or internal peel – Table 1). However, after a visual inspection of edges that correspond to tissue type, we observed differential co-expression of canopy position labels within clusters where the internal vs. external samples appeared to have different means and overlapping variance within a given tissue-specific cluster, but were apparently not sufficiently distinct for the GMMs to be able to differentiate them. We refer to edges from these clusters as potential “phased” edges. Therefore, to identify phased edges, we performed a Hotelling *t*-test (Hotelling, 1931); alpha was set to 0.001. The performHotellingTest function was created to do this and added to the KINC.R package at^9^. The edges with significant phasing were retained in the final tissue specific co-expression networks.

**TABLE 1:** A large majority of condition-specific edges (hence co-expressed) are tissue specific.

Condition	Number of edges	Number of genes
Peel	27,532	800
Cortex	6,442	581
External peel	35	56
Internal peel	66	95
External cortex	19	34
Internal cortex	20	32
External	1	2
Internal	2	4

*A minority are canopy-by-position specific. Genes can have multiple edges.*

### Module Discovery

Modules within the csGCNs were discovered using the KINC.R package (see text footnote 9), a supplemental R package of KINC, using the findLinkedCommunities function which wraps the linkcomm edge-based module discover tool (Kalinka and Tomancak, 2011). The function finds modules for disjoint subgraphs separately, was instructed to merge smaller modules into larger meta modules and use the “mcquitty” argument for hierarchical clustering. Module merging thresholds of 0.3 for peel and 0.6 for cortex resulted in 21 and 6 modules, respectively (Supplementary File 3). GO term enrichment analysis of modules was performed using the same methods as described above for the DE analysis and the code used for this analysis is included in Supplementary File 2.

## Results

### Fruit Quality Analyses

Pears picked from internal and external canopy positions showed an overlapping distribution of *I*_*AD*_ and were sorted into 5 classes to afford finer-grained comparisons (Figure 1). Class C (1.80–1.89 *I*_*AD*_) had a similar proportion of pears for both internal and external positions (respectively, 27.0 and 23.4%). At all time points, external fruit had blush (diffuse red) overcolor ranging from average 6.7 to 11.9%, while internal fruit had no overcolor (Table 2). While the chroma (saturation) of fruit background color did not differ between internal and external pears, hue angle did show significant differences. External fruit had a higher (yellower) hue compared with internal fruit that appeared more green. These differences in peel color were found in class C fruit at all time points except after 6 months storage. Only after 8 months of storage did internal fruit have a lower lightness of the background color compared with external fruit.

**TABLE 2:** Fruit color characteristics differed by canopy position with in *I*_*AD*_ class C.

Canopy position	Months Storage	Blush Overcolor%	Background color				
			L*	a*	b*	Hue	Chroma
External	**0**	6.65 A	60.75	−15.75	37.78	112.5 B	41.0
Internal		0.00 B	56.68	−16.30	35.64	115.1 A	39.2
External	**3**	7.19 A	67.70	−14.83 A	40.65	110.0 B	43.3
Internal		0.00 B	67.44	−15.82 B	40.69	111.3 A	43.7
External	**6**	10.13 A	68.29	−14.38	41.51	109.1	43.9
Internal		0.00 B	68.12	−14.99	41.06	110.0	43.7
External	**8**	11.93 A	69.3 A	−13.58 A	41.63	108.1 B	43.8
Internal		0.00 B	67.3 B	−15.19 B	41.54	110.1 A	44.2

*Post hoc SNK test discriminated the means and was reported with capital letters by position within each timepoint. Blush overcolor is diffuse redness of the peel. **L**, lightness (0 = black, 100 = white), a^∗^, green–red, b^∗^, blue–yellow. n ≥ 14 fruit. P < 0.05.*

External pears in class C had a higher at-harvest average fruit weight compared with internal fruit with differences up to 37.8 g; this difference was not significant at 6 months of storage (Table 3). After both 3 and 8 months of storage the fruit weight reflected the same differences as at harvest. *I*_*AD*_ at harvest was not significantly different (both internal and external fruit mean was 1.85), but at both 3 and 6 months of storage, the average *I*_*AD*_ values differed significantly, with internal class C pears showing higher value (less ripe) than external pears. SSC within class C were higher in external fruit compared to internal fruit, with a range of significant differences after ripening from 1.1 to 1.9°Brix after 7 days of ripening (following both 3 and 6 months of storage). pH average value was highest for internal fruit at 3 months, but was not significantly different during subsequent months of storage.

**TABLE 3:** Fruit quality for *I*_*AD*_ class C reveal persistent differences between canopy positions.

Canopy position	Months storage	Weight (g) at harvest	Weight (g) in storage	Firmness in storage	Firmness + 7 days @ 25°C	*I*_*AD*_ In storage	SSC in storage	SSC + 7 days @ 25°C	pH
External	**0**	259.8 A		62.4 A			14.0 A		
Internal		222.0 B		59.7 B			12.2 B		
External	**3**	247.7 A	243.5 A	61.1 Aa	9.8 Bb*	1.78 B	13.9 A	14.4 A	3.85 B
Internal		214.8 B	210.7 B	57.2 Ba	15.2 Ab*	1.81 A	12.7 B	13.3 B	3.96 A
External	**6**	247.7	241.1	57.0 a	9.6 b	1.71 B	14.0 Ab	15.4 Aa	3.73
Internal		234.6	228.3	55.1 a	11.0 b	1.76 A	12.3 Bb	13.5 Ba	3.65
External	**8**	257.7 A	249.9 A	50.5 a	9.7 Bb	1.63	14.2 A	14.2	3.71
Internal		227.2 B	220.7 B	52.3 a	11.3 Ab	1.67	12.6 Bb	13.7 a	3.66

*SSC, soluble solids content (or °BRIX), bold ^∗^ P < 0.05 for change in firmness in response to conditioning. Post hoc SNK test discriminated the means and was reported with capital letters by position within each timepoint (vertically) and in small letters within each position × timepoint between unripe and ripe (horizontally). I_AD_ at harvest showed no significant differences. n ≥ 14 fruit. P < 0.05.*

Class C external fruit showed larger changes in fruit firmness in response to ripening than internal canopy fruit. This difference was the largest after 3 months of cold storage (bold in Table 3). This result is consistent with the analysis of the larger (non-*I*_*AD*_-classified) fruit data set (Serra et al., 2018). This difference of ripening capacity suggests different at-harvest maturity for these samples (despite similarity of *I*_*AD*_ measurements) – fruit of more advanced maturity showed a larger change in fruit textural quality (for which firmness is a proxy). Subsequent transcriptomic analyses were therefore conducted using fruit in *I*_*AD*_ class C.

### Transcriptome Analysis of Fruit From *I*_*AD*_ Class C

Generally, RNA-seq data were of high quality as >99.5% of the read data passed quality filtering and trimming. On average 63.8% of the ‘d’Anjou’ RNA-seq data mapped to BartlettDH v2.0 (Linsmith et al., 2019) gene models (mRNAs). A principle components analysis (Supplementary Figure 1) of our pilot GEM (peel data) revealed that the transcriptome data had sufficient structure to distinguish fruit based on canopy position and tissue, thus providing contrasts to explore molecular signatures of differential ripening between canopy positions, as well as search for possible biosignatures. We selected genes for qPCR validation following Hargarten et al. (2018) and the validation showed an average *R*^2^ of 0.72 for the linear regression of all independent biological replicates (*R*^2^ = 0.83 for means) across a subset of samples for the each of the 10 candidate genes (Supplementary Table 1).

**FIGURE 2:**
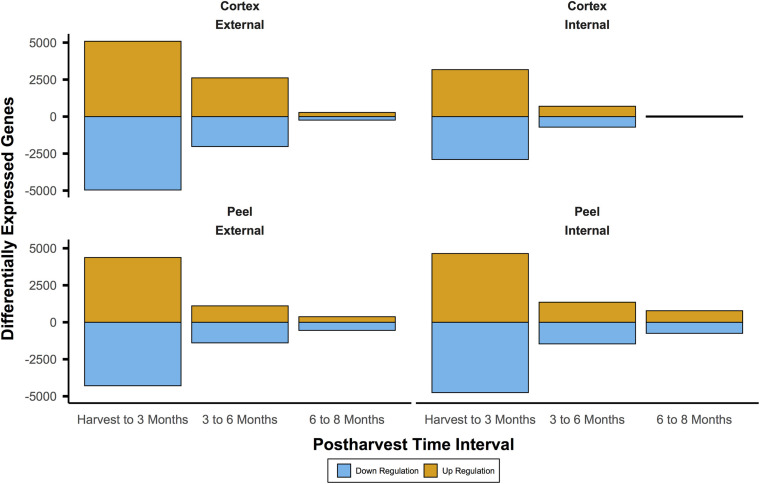
The number of differentially expressed genes was initially large and diminished through time. This pattern indicates large shifts of expression during the first three months of controlled atmosphere storage at −0.5°C may be involved in ripening processes. This includes gain of the chilling-induced capacity to ripen for European pear varieties like ‘d’Anjou’. It also shows that massive shifts in gene expression may obscure useful biosignatures. Significant differential expression was determined by Bonferroni corrected *P* < 0.05.

**FIGURE 3:**
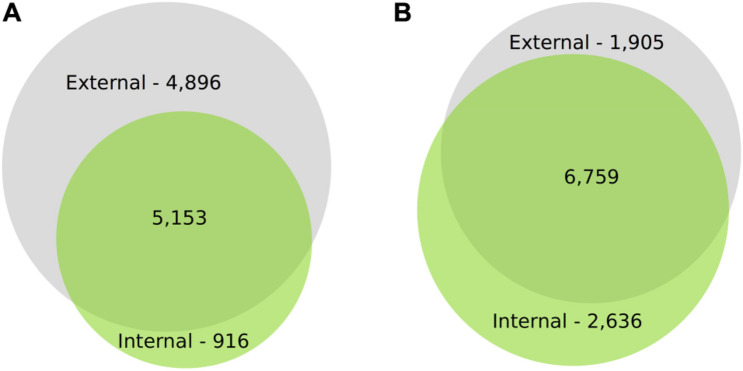
There is substantial overlap in DEGs through time for cortex and peel. Proportional VENN Diagrams (www.biovenn.nl) showing: **(A)** Differential gene expression of the cortical tissues at 3m from internal fruit is largely a subset of differentially expressed gene from external canopy positions, **(B)** Differential gene expression of the peel tissues at 3m from internal fruit is proportional to external fruit, with substantial overlap. This suggests that the contrasting light environment of the two canopy positions has a larger effect on peel tissues, yet despite significant fruit flesh quality differences, DEGs from internal cortex is largely a subset of external DEGs. Significant differential expression was determined by Bonferroni corrected *P* < 0.05.

For *I*_*AD*_ class C, the number of DEGs through time for both canopy position and tissues (e.g., internal peel) were initially large and decreased at each time point from harvest through CA storage (Figure 2). Across all samples, after 3 months of storage 15,353 genes (with substantial overlap – Supplementary Figure 2) had significant DE compared to at-harvest levels of expression. At subsequent time points, 7,183 genes from 3 months to 6 months, and 2,425 genes from 6 months to 8 months were DE (Figure 2). The DEGs in pear fruit cortex from internal canopy positions were largely a subset of DEGs in the cortex of fruit from external canopy positions (Figure 3A) with less than 10% of DEGs (both up- and downregulated) being unique to internal canopy position fruit. For peel tissue the differences were proportional with substantial overlap (Figure 3B). These trends continued for subsequent time points (Supplementary Figure 3).

**FIGURE 4:**
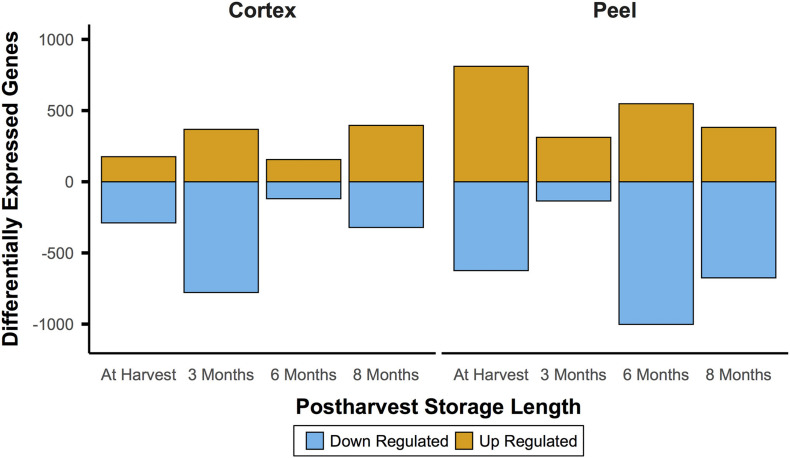
Gene expression between canopy positions suggests a persistent effect of phased at-harvest fruit maturity. Gene expression for both tissue types was of a fairly consistent magnitude throughout the experiment, which spanned 8 months of controlled atmosphere storage at −0.5°C. This contrasts with the initially large and waning magnitude of patterns of differential expression through time in storage, and indicates the possibility that gene expression signatures may be useful to predict fruit quality at and after harvest. Significant differential expression determined by Bonferroni corrected *P* < 0.05.

In comparison to the very large number of DEGs through time for a given canopy position and tissue, comparisons *between* canopy positions for each tissue type (e.g., internal vs external peel) showed changes that were smaller by roughly an order of magnitude. For instance, at harvest, 1,612 genes between internal and external tissues were differential. However, unlike the decreasing number of DEGs through time, the number of DEGs between equivalent fruit from different canopy positions remained relatively stable – 1,575 DEGs at T1, 1,737 DEGs at T2, and 1,590 DEGs at T3 (Figure 4). However, there was only a single DEG across all time points (pycom01g10280, a putative chaperone binding protein).

Comparisons were made to recent studies that examined expression of some relatively well-characterized climacteric fruit ripening genes, ACO, ACS, and PG, photosystem genes and others (Ziosi et al., 2008; Busatto et al., 2019). In general our results agreed, though direct comparisons are scarce since sampling for gene activity many months into the postharvest period has not been done extensively (Supplementary File 4). While many of these genes had patterns in our experiment that were concordant with the previous work (i.e., for at-harvest gene expression), they were generally not statistically significant in our analysis, most probably due to the fine contrast in our fruit samples.

### Functional Enrichment of DE Genes

The most significant enriched functional terms in our DE analysis (Supplementary File 1) correspond to differences in the canopy light environment–in the peel of external canopy fruit we see a strong enrichment (generally *P* < 0.001) at harvest of light harvesting and photosynthesis related GO terms in genes that are differentially upregulated. Among down regulated external peel genes at harvest was an enrichment of GO terms including some related to ethylene perception, DNA binding and transcription factor activity, and, still consistent with a contrast of light environment, light sensing and photoperiodism related terms.

Cortical tissues showed GO term enrichment among DE upregulated genes of terms for binding to cytoskeletal elements, intracellular transport, vesicle formation and docking, and many related to mitotic spindle assembly. We observed highly significant (*p* < 1e−20) enrichment for the GO biological process (BP) term “translation”, GO molecular function (MF) term “structural constituent of ribosome”, and GO cellular compartment (CC) term “ribosome” among GO terms for differentially downregulated genes in external cortical tissues after 3 months of storage. However, the relative contribution of these genes, or the many hundreds of other genes with enriched GO terms, to the different fruit quality parameters remains unclear.

### Hints of Functionally Related Shifts in Gene Expression Through Time

Many of the above enriched functional terms are also seen in other comparisons of fruit tissues from different canopy positions or time points, though the genes and specific terms are not always the same (Supplementary File 1). For instance, 40 genes with GO molecular function (MF) term “microtubule binding” are upregulated from harvest to 3 months of storage in cortical tissue of external fruit. Then, in a comparison of fruit cortex *between* canopy positions after 3 months of storage, two differentially upregulated genes from the external fruit have the GO BP terms “microtubule nucleation”, and the GO CC terms “spindle pole” and “microtubule organizing center”, and a third gene has the term “HAUS complex”, a protein complex that regulates microtubule organization in plants (Goshima et al., 2008; Lawo et al., 2009; Liu et al., 2014). Two others have the GO BP term “spindle assembly”. These terms may indicate successive changes of gene expression in related pathways or processes that persist through storage, but do not result in persistent differential regulation of individual genes across the protracted postharvest period.

### Machine Learning, Co-expression, and Tests for Phased Gene Expression

To add to the DEG analysis and identify potential relationships and the conditions in which those genes interact, we performed csGCN analysis. Creation of csGCNs can be time consuming with large sample sizes. Therefore, to limit the number of genes for creation of the network we selected genes that were most predictive. This was performed using a modified random forest approach (via Boruta implemented in GSForge (see text footnote 7)) to find gene activity that was associated with canopy position and tissue type, and resulted in a list of 830 genes that had a feature importance score above their corresponding scores from randomized iterations of the input data. Of these, nearly all (799, 96.3%) showed condition specific co-expression (i.e., network edges) between nodes (genes) in subsequent network analyses. However, when we looked for edges in subgraphs that were specific to canopy position, i.e., in cortex from external canopy fruit, we found that the majority of condition specific edges were tissue specific (Table 1). Subgraphs specific to canopy position were missing, yet our fruit quality analyses showed a clear effect of canopy position. Furthermore, our PCA suggests significant structure in the gene expression data, and we do observe persistent differential gene expression associated with canopy position.

**FIGURE 5:**
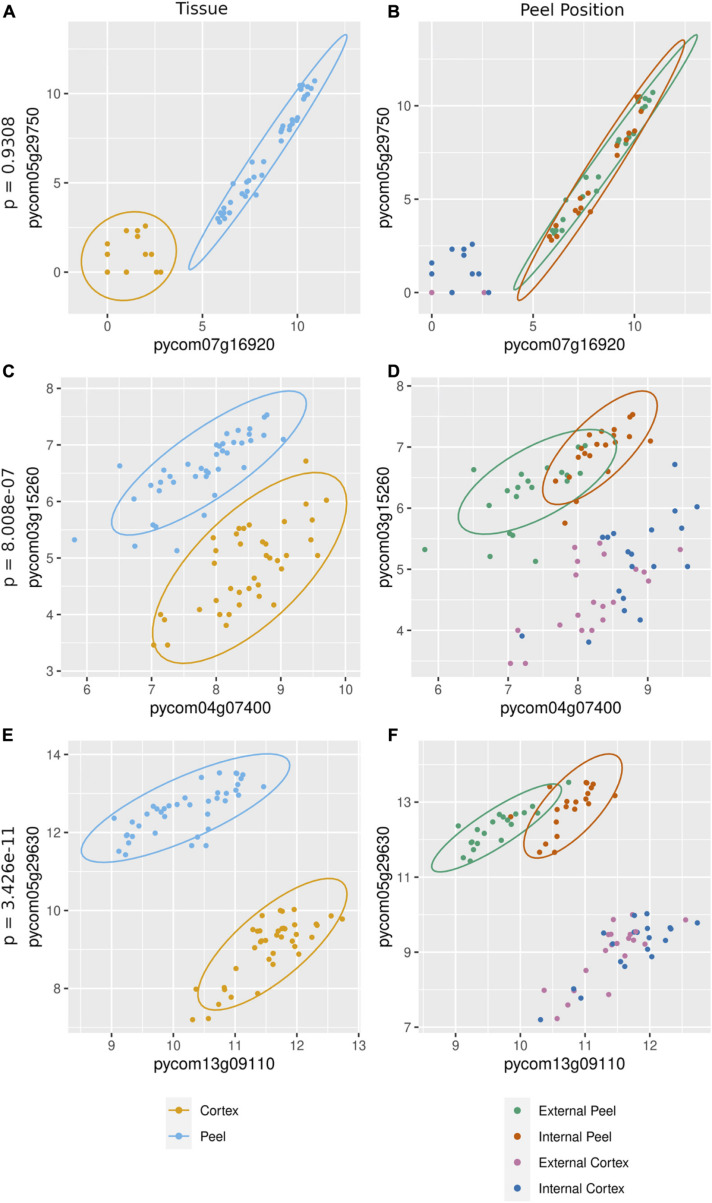
Gene expression in canopy specific samples can show significantly different means, indicative of phased co-expression. **(A,C,E)** – examples of co-expressed genes (i.e., an edge detected by KINC) – the orange cluster is for cortex expression, and the blue cluster is for peel expression. **(B,D,F)** – tests for phasing in peel clusters (green for external peel, red for internal peel) that shows no difference in means between canopy positions **(B)**, and two examples of significant difference in means by canopy position **(D,F)**. Our sorting scheme created contrasts of fruit quality and ripening, derived from at-harvest differences in maturity. Phased gene expression patterns mirror the phased maturity we imposed on fruit harvest by sorting fruit by canopy position, suggesting that phased (i.e., differentially co-expressed) genes are involved in ripening.

**FIGURE 6:**
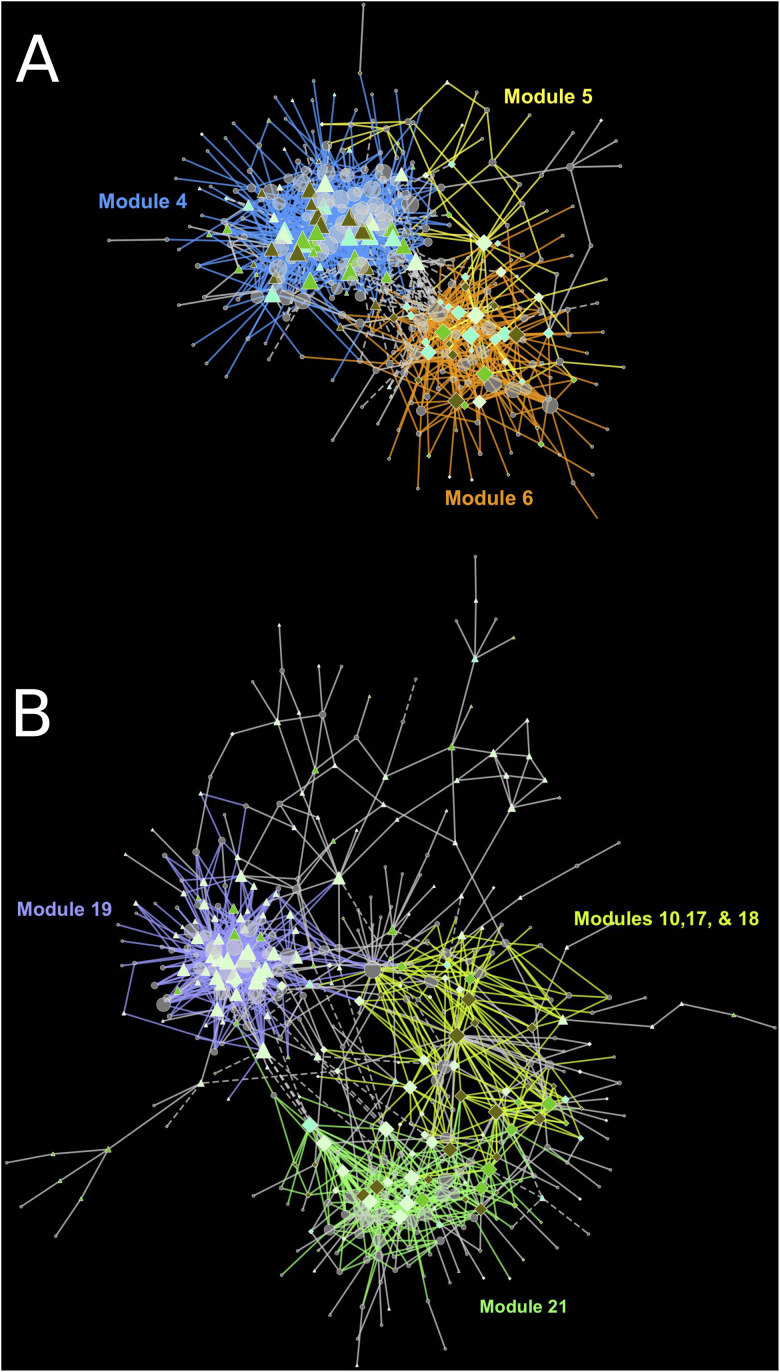
Tissue-specific, position-phased, csGCNs colored by modules and containing differentially up or down regulated genes. **(A)** cortex phased co-expression network, **(B)** peel phased co-expression network. Triangles indicate upregulation in external canopy positions, diamonds indicate downregulation. Node color intensity indicates DE at different timepoints with darker colors showing differential expression at later timepoints. Significant differential expression was determined by Bonferroni *P* < 0.05. Colored edges and matching labels indicate major modules. Gray edges indicate minor modules with a small number of edges. See Supplementary File 3 for network files.

The cause of missing position specific csGCNs was a result of a high degree of overlap in expression of canopy- and tissue-specific gene expression as demonstrated in the examples of Figure 5. In those cases, the variation due to position fell within that of tissue and often appeared phased (the mean and variance of each position was distinctly shifted) and hence default KINC processes could not distinguish differences in position. Therefore, we added additional functionality to KINC (via the KINC.R supplemental R package) that searched for phased gene activity by testing for differences in the mean of canopy specific samples within a tissue class. This resulted in discovery of tissue-specific position-phased csGCNs. Within these csGCNs we found hundreds of co-expressed genes (339 from cortex and 442 from peel) that showed a significant difference in means, suggesting widespread phased gene expression among genes selected by GSForge. Next, we performed edge-based module discovery that allows genes to be represented as multifunctional (i.e., in different modules) and that circumscribes edges into modules of highly interacting genes. This resulted in 6 modules in the phased cortex csGCN and 21 in the phased peel csGCN. Resulting csGCNs are shown in Figure 6 with edges colored by modules. Additionally, genes that were significantly differentially expressed (i.e., equivalent tissues from different canopy positions) are highlighted in Figure 6. We found that co-expressed genes with position-phased expression nearly exclusively contained genes that were consistently higher (Figure 6 – triangles), or lower (Figure 6 – diamonds) in external canopy fruit. Many modules showed successive DE through time (Figure 6 – indicated by node colors).

**FIGURE 7:**
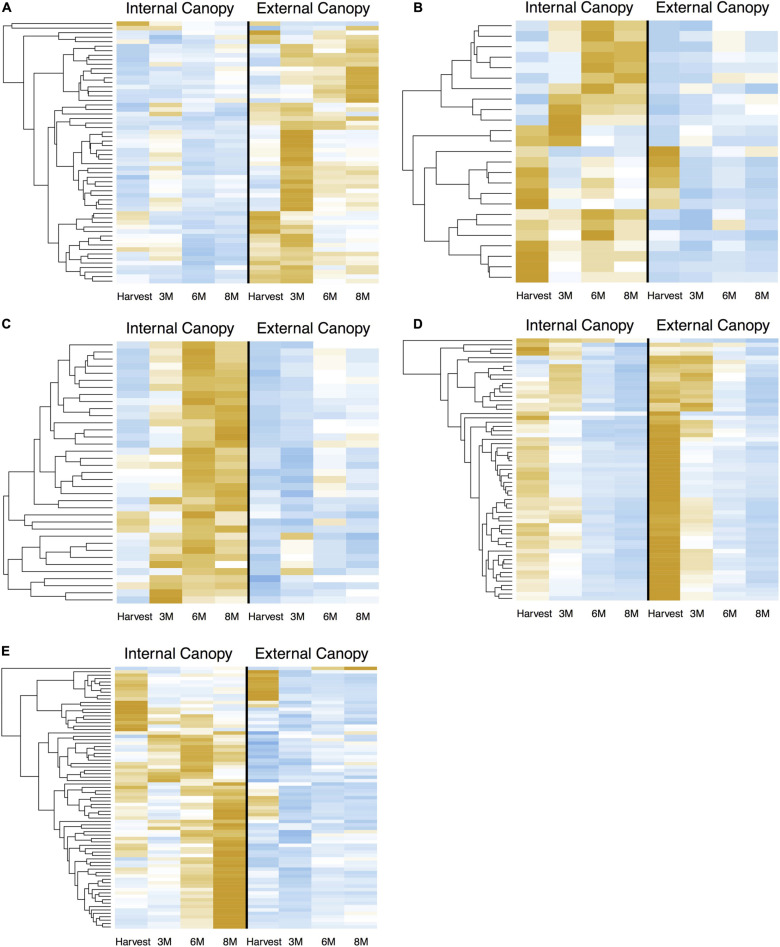
Successive shifts of gene expression that are associated with differential postharvest outcomes are apparent in csGNC modules from *I*_*AD*_ class C. DEGs in **(A)** cortex module 4, **(B)** cortex module 5, **(C)** cortex module 6, **(D)** peel module 19, **(E)** peel module 10, 17, 18, and 21. Orange colors indicate higher relative expression, blue indicates lower relative expression. Plots show Z-score transformation of normalized (RPKM) counts and are independently scaled. All features are significantly differential (Bonferroni *P* < 0.05) at one or more timepoints between equivalent canopy positions and are part of the indicated co-expression module. Postharvest outcomes in our experiment indicate fruit within *I*_*AD*_ class C from external canopy positions are more mature. csGCN modules therefore contain DEGs that are associated with these different postharvest fruit quality characteristics. In addition to offering clues about the genetic control of ripening, it also suggests the possibility to use gene-expression based biosignatures to predict future fruit quality, including the capacity to ripen. DEGs are listed in Supplementary File 3.

When we examined the expression of DEG in cortex module 4 (Figure 7A) across all time points, we see successive shifts of expression in external canopy fruit that are echoed in the internal canopy fruit (visible when scaled independently – see Supplementary Figure 4). The pattern is similar in cortex modules 5 and 6, except that gene expression is generally lower in external canopy fruit (Figures 7B,C). For peel tissues, gene expression in module 19 tended to be highly distinctive at harvest and after 3 months, but was convergent thereafter (Figures 7D, 6B). For co-expressed and phased peel genes that showed lower expression in external fruit peel tissues, modules 10, 17, 18, and 21 captured the majority of DE genes and were grouped for comparison. In this grouped set, the trends were more similar to cortex module 5 and 6, with a successive character (Figure 7E).

### Functional Enrichment Analysis of Co-expression Modules

A functional enrichment analysis of csGCN modules revealed highly distinctive profiles (Supplementary File 1). One of particular interest was observed in the phased peel network module 19 (Figure 6). The GO BP term “farnesyl diphosphate biosynthetic process” was enriched (*P* = 0.017) among genes in the module that showed a higher expression in the peel of external fruit. In cortical tissues, this term was enriched among *down* regulated genes in the cortex network (Figure 6 – module 6). Farnesyl diphosphate is a precursor to alpha-farnesene (Gapper et al., 2006), a metabolite associated with the pome fruit peel disorder superficial scald (Whitaker et al., 2009).

The functional profiles of other csGCN modules include possible links to the epigenetic machinery recently reported by the fruitENCODE project as crucial for control of the ethylene climacteric [Lu et al. (2018)^10^ ]. In both peel module 21 and cortex module 4 the GO BP term “nucleosome assembly” (respectively, *P* = 0.004 and *P* = 0.014) was significantly enriched as was the GO CC term “nucleosome” (respectively, *P* = 0.006 and *P* = 0.034). In cortex module four the GO CC term “FACT complex (facilitates chromatin transactions)” was enriched (*P* = 0.032), and in peel module 21 the GO BP terms “histone lysine methylation” (*P* = 0.049) and the GO CC term “DNA topoisomerase type II (ATP-hydrolyzing)” (*P* = 0.004) were enriched.

In the peel network the two genes annotated with the GO CC term “nucleosome” showed lower expression in external fruit, one was pycom15g21750, which is a H1-like histone protein [a possible winged-helix type transcription factor – see annotation summary at (see text footnote 9)]. The other peel network gene with the “nucleosome” term was pycom09g10570, a putative telomere repeat binding (TRB) factor. Among the top BLASTp hits to Arabidopsis proteins [see BLAST results at GDR (see text footnote 9)] for this gene was AtTRB1 (AT1G49950 – Araport BLASTp via GDR 2.3e-35). The significantly enriched GO BP term “nucleosome assembly” (*P* = 0.014) was also associated with two *up*regulated genes in external fruit from our cortex network (pycom12g22270 and pycom15g21750 in module 4 – both putative H1 – like histone proteins).

Another set of significantly enriched GO terms reflects the different light environment of the two canopy positions defined in this study. Among upregulated genes in the networks, we see several significantly enriched GO BP terms in peel network module 19 and cortex module 4 related to photosynthesis, including “photosynthesis” (peel mod. 19 *P* = 0.019), “photosynthesis, light harvesting” (peel mod. 19 and cortex mod. 4 *P* < 0.001), “photosystem I assembly” (peel mod. 19 *P* = 0.021, cortex mod. 4 *P* < 0.001) and “carotenoid biosynthesis” (peel mod. 19 *P* = 0.033), and the GO CC term “photosystem I reaction center” (peel mod. 19 *P* = 0.036).

Functional terms in modules containing genes that were downregulated in external fruit (Figure 6 – cortex modules 5 and 6, and peel modules 10, 17, 18, and 21) were for genes related to DNA damage detection and repair, meiotic nuclear division, and endoreduplication. These genes include a hypothetical protein (pycom02g05270) with the significantly enriched (cortex mod. 5 *P* < 0.001) GO CC term “SOSS complex”. The Sensor Of Single-Stranded DNA (SOSS) complex is involved in the G2/M cycle checkpoint where entry to mitosis is blocked when DNA damage is detected (Lobrich and Jeggo, 2007). The *P. communis* gene pycom05g16060 (‘‘male and female meiotic nuclear division’’ GO BP terms – cortex mod. 5 *P* = 0.009 and *P* = 0.028, respectively) is annotated as XRI1 which is possibly involved in meiotic DNA repair (see At548720 XRI1 at^11^). Finally, pycom01g23940, a putative double stranded DNA binding protein [BIN4 (see text footnote 9)] with the significantly enriched GO BP term “endoreduplication” is present in cortex module 6 (*P* = 0.047) and peel module 21 (*P* = 0.043).

## Discussion

### Canopy Position-Dependent Fruit Quality Differences

The comparison between pears harvested from external and internal tree canopy positions belonging to *I*_*AD*_ class C at each postharvest time point revealed some significant differences in terms of color and appearance. For instance, after storage (or also after a ripening period of 7 days), many parameters that are important for consumer preference including color, sugar content, and texture (Chen, 2016) were different between fruit from the two canopy positions. The differences in peel color, with external fruit appearing less green and more yellow, are consistent with previous work (Zhang et al., 2016; Serra et al., 2018). Moreover, in the study on the broader sample set (not sorted by *I*_*AD*_), levels of flavonol glycosides, carotenoid pigments, and thylakoid membrane components are associated with ‘d’Anjou’ peel of fruit harvested from external positions (Serra et al., 2018). The larger fruit size and higher SSC for external pears suggest some of the differences in quality and ripening by canopy position are ascribable to light exposure and the effect of light on fruit development and dry matter accumulation during the growing season (Cockcroft and Nair, 2012; Zhang et al., 2016; Goke et al., 2020).

While *I*_*AD*_ has been demonstrated as a useful proxy for maturity (Ziosi et al., 2008; Costamagna et al., 2013; Gagliardi et al., 2014; Vidoni et al., 2015), the highly heterogeneous growth environment of large, open-vase trees present challenges for this sorting method. Indeed, the fruit in *I*_*AD*_ class C was significantly different, while the *I*_*AD*_ values were not. The change in *I*_*AD*_ during long term CA storage was less for pears picked from the internal canopy position, suggesting slower kinetics of ripening for ostensibly less mature pears. Further, after 3 months of CA storage [during which time ‘d’Anjou’ pear acquires the capacity to ripen (Blankenship and Richardson, 1985)], the significant texture differences do indicate a difference due to fruit maturity at harvest, and thus greater ripening capacity for external fruit, despite no at-harvest differences in *I*_*AD*_. While the classification by *I*_*AD*_ did not discriminate maturity differences in this contrast, it did create an opportunity to dig deeper with a global-scale gene expression analysis. We therefore aimed to discover clues about these differences in maturity with regard to genes that control fruit ripening at various points during the postharvest period for ‘d’Anjou’ pear.

### Differential Gene Expression Survey

The massive shifts in gene expression that diminished in magnitude during long-term CA storage contrast with the relatively small-scale and stable differences between equivalent samples of external and internal canopy fruit. Together, this indicates that fruit maturity differences at harvest within *I*_*AD*_ class C resulted in persistent gene expression differences that were distinguishable from the larger background changes through time. However, the DEG profiles between canopy positions were distinctive at each time point, with only a single DEG across all time points. Because of the fine contrast that was imposed by picking fruit from the same tree, on the same day, and sorting the fruit into discrete classes based on *I*_*AD*_ and other criteria, we did not expect large gene expression differences between canopy positions within *I*_*AD*_ class C. These observations highlight a challenge of selecting genes that may explain postharvest differences in fruit quality–against the backdrop of massive shifts of gene activity during long-term storage of pears, informative biosignatures may be obscured. Further, through time in storage, DEGs in internal fruit tended to have a large degree of overlap with DEGs in external fruit, making the task of finding discriminatory signatures difficult.

We hypothesize that genes with expression patterns that distinguish canopy positions, especially in the cortical tissues and also earlier in the storage period where ripening was more divergent, may encode proteins that play an important role in acquisition of chilling-induced capacity to ripen. However, more work is needed to help decipher which of the ∼10,000 DEGs in external canopy cortical tissues during postharvest storage play a substantial role in the chilling-induced capacity to ripen. We did observe that fruit from external canopy positions showed a greater change in texture in response to a ripening period after 3 months of storage, the time during which chilling induced capacity to ripen was acquired (Blankenship and Richardson, 1985). That physiological difference was coincident with very strongly enriched functions among downregulated genes that encode translation machinery, and also with upregulated genes annotated with GO terms relating to cytoskeletal elements and intracellular transport machinery. For riper fruit that show greater changes in textural quality, this could indicate a switch away from translation to an increase of intracellular transport processes, but more work is needed. Follow-up work could include experiments with a higher density sampling earlier in the storage period (with an emphasis on fruit ripening capacity), and experiments that parse the fruit response to controlled atmospheres, cold storage, and the interaction of harvest maturity with various postharvest treatments.

Ultimately, the functional profile of genes that were differentially expressed across time points, especially the massive changes after 3 months of CA storage, are unlikely to be informative with regard to any specific process. This is due in part to the successive nature of metabolic and transcriptomic shifts during storage, documented on similar time scales in other tree fruit (Gapper et al., 2017). Indeed, the enriched functions from the ∼10,000 DE genes in fruit cortex are numerous and diverse (Supplementary File 1) making them difficult to parse in a meaningful way with regard to the observed fruit quality differences.

### Genes With Phased, Differential Co-expression Are Candidate Biomarkers

Our analysis reveals genes that have a tissue-specific, coordinated (co-expressed) and phased character that reflect differences in maturity of class C fruit at harvest. The character of expression also suggests that future postharvest tools based on gene expression (i.e., biosignatures) may need to target large expression modules that have changing gene membership throughout the postharvest period rather than individual genes. It may also be prudent to consider that different gene clusters may have utility at different times, depending upon when producers might assess fruit. As validation of candidate biomarkers progresses, ratios of gene activity between modules and through time may also provide greater precision and accuracy than tracking individual genes. Below we discuss examples that serve to confirm our approach, but also offer new clues about genes that influence postharvest pear fruit quality, and specifically the capacity to ripen.

### Biosynthesis Gene for a Metabolite Associated With Maturity-Linked Postharvest Disorder

As stated above, the metabolite alpha-farnesene is associated with the peel disorder superficial scald (Lurie and Watkins, 2012). The discovery of a differentially co-expressed gene related to the synthesis of a metabolite implicated in a maturity-linked postharvest disorder provides evidence that our approach may provide maturity-related biosignature genes. Further, external fruit from the larger sample set (Serra et al., 2018) had significantly more superficial scald than internal fruit, as well as higher levels of many metabolites, including conjugated trienols of farnesene and acylated steryl glycosides, associated with risk of developing superficial scald in apples and pears (Whitaker et al., 2009). Indeed, it has been shown that superficial scald risk is associated with pear fruit maturity (Calvo et al., 2019).

### Signatures for Epigenetic Regulation of Postharvest Fruit Quality

The nucleosome is the fundamental subunit of chromatin consisting of DNA wrapped around histone proteins, the state of which (e.g., methylation) serves as a basic layer in regulation of gene expression (Rudnizky et al., 2017). The FACT (facilitates chromatin transactions) complex is important for chromatin remodeling and normal methylation patterns in Arabidopsis (Frost et al., 2018), and type II DNA topoisomerases are abundant chromatin proteins that can influence gene expression (Roca, 2009). Mutations in TRB1 enhance the lhp1 mutant phenotype (see text footnote 11) which consists of numerous severe growth and development defects (Mateo-Bonmatí et al., 2018). Veluchamy et al. (2016) showed that LHP1 regulates the repressive epigenetic mark, H3K27me3, reported by Lu et al. (2018) to be critical for controlling the expression of genes involved in a positive feedback loop of ethylene signaling during ripening across many plant lineages (including *Pyrus*) in which the ethylene climacteric evolved independently.

It is, therefore, possible that genes with the enriched GO terms related to epigenetic machinery may be involved in guiding the epigenetic modifications known to repress positive ethylene feedback loops associated with ripening (Lu et al., 2018); however, their exact roles and why they appear to show opposite patterns of expression in peel and cortex of pear fruit are unclear. This suggests tissue specific patterns of epigenetic control of ripening and may also help explain the highly peel specific necrosis observed in superficial scald, which is a maturity-linked peel disorder of pear, and apples (Lurie and Watkins, 2012). Collectively, the genes with annotations related to epigenetic machinery are good candidates for studies aimed at further deciphering the molecular mechanisms that integrate cues from the production and postharvest environment with control of pear fruit ripening. This is especially important for European pears that require a predictable progression of ripening, namely textural changes in fruit flesh, to meet consumer expectations.

### Photosynthesis Genes Are Upregulated in External Fruit Tissues

Concordant with the enrichment of photosynthesis related genes among our putative biomarker genes is that metabolomic analysis of peel [from the larger sample set from which our samples were taken - described in Serra et al. (2018)] showed xanthophylls and B-carotene levels are elevated in peel of ‘d’Anjou’ pears harvested from external positions. Most of the upregulated peel network genes are only significant at harvest and after 3 months of storage, suggesting that some of the effects of the contrasting light environment in the different canopy positions may not persist throughout storage. Contrasts of fruit maturity that are independent of the light environment are needed to clarify the role of these genes as they relate to fruit maturity, especially with regard to molecular mechanisms that link the fruit production environment and genetic control of the ethylene production associated with climacteric ripening.

### Downregulated Genes in More Mature Fruit Offer New Clues About the Mechanisms of Ripening

It is possible that the mechanisms for endoreduplication may tie together unexpected functional enrichment signatures involving cell cycle regulation and meiosis that are observed in fruit tissue samples that are not likely to contain dividing cells. It is known that endoreduplication is involved in the specification of organ size (Bhosale et al., 2019), and horticultural maturity for pears coincides with the end of fruit cell expansion. Further, the process of endoreduplication involves cell division machinery, namely that related to DNA replication (Bhosale et al., 2019; Qi and Zhang, 2019). In tomato, endoreduplication has been shown to be important for fruit development, and Bourdon et al. (2012) showed that increased transcription was due to endopolyploidy. In fact, among downregulated genes in the network modules listed above, the significantly enriched GO BP terms “regulation of transcription, DNA-templated” and “regulation of DNA-templated transcription, elongation” co-occurred with the genes discussed above that may relate to endoreduplication. All together, these suggest a role for endoreduplication in the control of fruit maturity or capacity to ripen, as downregulation of these genes (which were also co-expressed and phased) were associated with fruit of more advanced maturity.

### Perspectives on Biosignatures for Predicting Ripening in ‘d’Anjou’ Pear

The molecular mechanisms that control European pear fruit maturity and ripening, indeed all pome fruits, are poorly understood. This is due in part to the intractability of genetic assays in Rosaceous fruit trees, but also the complex nature of ripening processes. Furthermore, variable production practices, a highly artificial postharvest period with multiple layers of postharvest technology, and narrow targets for fruit quality standards make experiments that aim to understand these mechanisms with regard to industry-relevant outcomes a persistent challenge. This work aimed to generate hypotheses about the molecular mechanisms behind differential postharvest ‘d’Anjou’ pear fruit quality.

We found that changes in the transcriptome during early months of storage were massive, and while these contained confirmatory gene activity signatures for known ripening genes, these were generally insufficient to distinguish fruit in our experiment. We dug deeper, using a novel combination of bioinformatics approaches to circumscribe hundreds of genes that had a subtle but significant phased character which mirrored the phased maturity that was experimentally imposed on the fruit at harvest. As our hypotheses are tested and we deepen our understanding of the genetic control of fruit quality, gene candidates that might be deployed as biosignatures will come into focus. For instance, a gene expression-based maturity index could allow a producer to estimate the proportion of fruit that have the capacity to ripen across lots. This information could be used to inform marketing decisions, leading to less food wastage and potentially reducing cost by more precise and efficient use of postharvest technology.

The next steps include additional years of data, fruit quality contrasts that are developed in multiple ways, and extensive validation that explores how these genetic factors respond to the production environment and horticultural practices, storage, and distribution. Fortunately, the catalog of horticulture research is large and offers many opportunities to explore well documented effects of production practices and postharvest technology with new global scale technologies and novel analytical methods. As our understanding of the genetic control of ripening improves, so do the tools available to manage pear fruit for enhanced quality.

## Data Availability Statement

The datasets presented in this study can be found in online repositories. The names of the repository/repositories and accession number(s) can be found below: NCBI BioProject ID PRJNA715928.

## Author Contributions

LH, SS, SM, DR, JM, and CP conceived the work. LH, HH, JH, SS, SF, and EW conducted the experiments. LH, HH, JH, SF, SS, and EW contributed analyses and interpretation. LH, HH, JH, SS, SM, SF, and JM wrote the manuscript. All authors contributed to the article and approved the submitted version.

## Conflict of Interest

The authors declare that the research was conducted in the absence of any commercial or financial relationships that could be construed as a potential conflict of interest.

## Footnotes

1 www.illumina.com

2 www.ncbi.nlm.nih.gov/sra

3 www.rosaceae.org

4 www.bioinformatics.babraham.ac.uk

5 www.qiagen.com

6 https://gitlab.com/R_packages

7 https://github.com/SystemsGenetics/GSForge

8 https://scikit-learn.org/

9 https://github.com/SystemsGenetics/KINC.R

10 http://www.epigenome.cuhk.edu.hk

11 www.arabidopsis.org

## References

[B1] Alexa A. Rahnenfuhrer J. (2019). topGO: enrichment analysis for gene ontology. *BioConductor*

[B2] Altman N. Krzywinski M. (2017). Clustering. *Nat. Methods* 14 545–546. 10.1038/nmeth.4299 PMC5905345 29664466

[B3] Argenta L. C. Fan X. Mattheis J. P. (2003). Influence of 1-methylcyclopropene on ripening, storage life, and volatile production by d’Anjou cv. pear fruit. *J. Agric. Food Chem.* 51 3858–3864. 10.1021/jf034028g 12797756

[B4] Awad M. A. Wagenmakers P. S. de Jager A. (2001). Effects of light on flavonoid and chlorogenic acid levels in the skin of ‘Jonagold’ apples. *Sci. Hortic.* 88 289–298. 10.1016/s0304-4238(00)00215-6

[B5] Bai J. Prange R. K. Toivonen P. A. (2020). “Pome Fruits”, in *Controlled and Modified Atmospheres for Fresh and Fresh-cut Produce*, eds Gil M. I. Beaudry R. M. (Boca Raton, FL: Academic Press), 267–286.

[B6] Bhosale R. Maere S. De Veylder L. (2019). Endoreplication as a potential driver of cell wall modifications. *Curr. Opin. Plant Biol.* 51 58–65. 31071565 10.1016/j.pbi.2019.04.003

[B7] Blanckenberg A. Muller M. Theron K. I. Crouch E. M. Steyn W. J. (2016). Harvest maturity and ripeness differentially affects consumer preference of ‘Forelle’, ‘Packham’s Triumph’ and ‘Abate Fetel’ pears (*Pyrus communis* L.). *Sci. Hortic.* 207 131–139. 10.1016/j.scienta.2016.05.012

[B8] Blankenship S. Richardson D. (1985). Development of ethylene biosynthesis and ethylene-induced ripening in’d’Anjou’ pears during the cold requirement for ripening. *J. Am. Soc. Hortic. Sci.* 10 520–523.

[B9] Bolger A. M. Lohse M. Usadel B. (2014). Trimmomatic: a flexible trimmer for Illumina sequence data. *Bioinformatics* 30 2114–2120. 10.1093/bioinformatics/btu170 24695404 PMC4103590

[B10] Bourdon M. Pirrello J. Cheniclet C. Coriton O. Bourge M. Brown S. (2012). Evidence for karyoplasmic homeostasis during endoreduplication and a ploidy-dependent increase in gene transcription during tomato fruit growth. *Development* 139 3817–3826. 10.1242/dev.084053 22991446

[B11] Busatto N. Farneti B. Tadiello A. Oberkofler V. Cellini A. Biasioli F. (2019). Wide transcriptional investigation unravel novel insights of the on-tree maturation and postharvest ripening of ‘Abate Fetel’ pear fruit. *Hortic. Res.* 6:32. 10.1038/s41438-018-0115-1 30854209 PMC6395599

[B12] Calvo G. (2002). Effect of 1-methylcyclopropene (1-MCP) on pear maturity and quality. *Int. Hortic.* 628 203–211.

[B13] Calvo G. Candan A. Larrigaudière C. (2019). ‘Packham’s Triumph’ superficial scald susceptibility is affected by maturity at harvest. *Acta Hortic.* 1256 135–142.

[B14] Chagne D. Crowhurst R. N. Pindo M. Thrimawithana A. Deng C. Ireland H. (2014). The draft genome sequence of European pear (*Pyrus communis* L. ‘Bartlett’). *PLoS One* 9:e92644. 10.1371/journal.pone.0092644 24699266 PMC3974708

[B15] Chen P. Varga D. (1997). “Determination of optimum controlled atmosphere regimes for the control of physiological disorders of ‘d’Anjou’pears after short-term, mid-term or long-term storage”, in *Proceedings of the 7th International Controlled Atmosphere Research Conference*, ed. Mitcham E. J. (Davis, CA: University of California).

[B16] Chen P. M. (2016). “Pear”, in *The Commercial Storage of Fruits, Vegetables, and Florist and Nursery Stocks*, eds Gross K. C. Wang C. Y. Saltveit M. E. (Washington, DC: United States Department of Agriculture, Agricultural Research Service), 471–480.

[B17] Cockcroft D. W. Nair P. (2012). Methacholine test and the diagnosis of asthma. *J. Allergy Clin. Immunol.* 130:556. 10.1016/j.jaci.2012.05.050 22743302

[B18] Costamagna F. Giordani L. Costa G. Noferini M. (2013). Use of ad index to define harvest time and characterize ripening variability at harvest in ‘Gala’ apple. *Acta Hortic.* 998 117–123. 10.17660/ActaHortic.2013.998.12

[B19] DeLong J. Prange R. Harrison P. Nichols D. Wright H. (2014). Determination of optimal harvest boundaries for Honeycrisp^TM^ fruit using a new chlorophyll meter. *Can. J. Plant Sci.* 94 361–369. 10.4141/cjps2013-241

[B20] Fan X. Mattheis J. P. Fellman J. K. (1998). Responses of apples to postharvest jasmonate treatments. *J. Am. Soc. Hortic. Sci.* 123 421–425. 10.21273/jashs.123.3.421

[B21] Faragher J. D. Brohier R. L. (1984). Anthocyanin accumulation in apple skin during ripening: regulation by ethylene and phenylalanine ammonia-lyase. *Sci. Hortic.* 22 89–96. 10.1016/0304-4238(84)90087-6

[B22] Fideghelli C. (2007). “Origine ed evoluzione”, in *Il Pero*, ed. Angelini R. (Milano: Bayer Crop Science).

[B23] Frost J. M. Kim M. Y. Park G. T. Hsieh P.-H. Nakamura M. Lin S. J. (2018). FACT complex is required for DNA demethylation at heterochromatin during reproduction in *Arabidopsis*. *Proc. Natl. Acad. Sci. U.S.A.* 115 E4720–E4729. 29712855 10.1073/pnas.1713333115 PMC5960277

[B24] Gagliardi F. Serra S. Ancarani V. Bucci D. Piccinini L. Noferini M. (2014). Preliminary results on Cv. ‘Abbé Fétel’ productivity and fruit quality in relation to tree architecture. *Acta Hortic.* 1058 151–158. 10.17660/ActaHortic.2014.1058.16

[B25] Gapper N. E. Bai J. Whitaker B. D. (2006). Inhibition of ethylene-induced α-farnesene synthase gene PcAFS1 expression in ‘d’Anjou’pears with 1-MCP reduces synthesis and oxidation of α-farnesene and delays development of superficial scald. *Postharvest Biol. Technol.* 41 225–233.

[B26] Gapper N. E. Hertog M. Lee J. Buchanan D. A. Leisso R. S. Fei Z. (2017). Delayed response to cold stress is characterized by successive metabolic shifts culminating in apple fruit peel necrosis. *BMC Plant Biol.* 17:77. 10.1186/s12870-017-1030-6 28431510 PMC5399402

[B27] Goke A. Serra S. Musacchi S. (2020). Manipulation of fruit dry matter via seasonal pruning and its relationship to ‘d’Anjou’ pear yield and fruit quality. *Agronomy* 10 1–23. 10.3390/agronomy10060897

[B28] Goshima G. Mayer M. Zhang N. Stuurman N. Vale R. D. (2008). Augmin: a protein complex required for centrosome-independent microtubule generation within the spindle. *J. Cell Biol.* 181 421–429. 18443220 10.1083/jcb.200711053 PMC2364697

[B29] Hargarten H. Waliullah S. Kalcsits L. Honaas L. A. (2018). Leveraging transcriptome data for enhanced gene expression analysis in apple. *J. Am. Soc. Hortic. Sci.* 143 333–346. 10.21273/jashs04424-18

[B30] Honaas L. Kahn E. (2017). A practical examination of RNA isolation methods for European pear (*Pyrus communis*). *BMC Res. Notes* 10:237. 10.1186/s13104-017-2564-2 28662720 PMC5492931

[B31] Honaas L. A. Hargarten H. L. Ficklin S. P. Hadish J. A. Wafula E. dePamphilis C. W. (2019). Co-expression networks provide insights into molecular mechanisms of postharvest temperature modulation of apple fruit to reduce superficial scald. *Postharvest Biol. Technol.* 149 27–41. 10.1016/j.postharvbio.2018.09.016

[B32] Honaas L. A. Wafula E. K. Wickett N. J. Der J. P. Zhang Y. Edger P. P. (2016). Selecting superior *de novo* transcriptome assemblies: lessons learned by leveraging the best plant genome. *PLoS One* 11:e0146062. 10.1371/journal.pone.0146062 26731733 PMC4701411

[B33] Hotelling H. (1931). The generalization of student’s ratio. *Ann. Math. Stat.* 2 360–378. 10.1214/aoms/1177732979

[B34] Jajo A. Rahim M. A. Serra S. Gagliardi F. Jajo N. K. Musacchi S. (2014). Impact of tree training system, branch type and position in the canopy on the ripening homogeneity of ‘Abbé Fétel’ pear fruit. *Tree Genet. Genomes* 10 1477–1488. 10.1007/s11295-014-0777-2

[B35] Jung S. Lee T. Cheng C. H. Buble K. Zheng P. Yu J. (2019). 15 years of GDR: new data and functionality in the genome database for Rosaceae. *Nucleic Acids Res.* 47 D1137–D1145. 10.1093/nar/gky1000 30357347 PMC6324069

[B36] Kalinka A. T. Tomancak P. (2011). linkcomm: an R package for the generation, visualization, and analysis of link communities in networks of arbitrary size and type. *Bioinformatics* 27 2011–2012. 10.1093/bioinformatics/btr311 21596792 PMC3129527

[B37] Kursa M. B. Jankowski A. Rudnicki W. R. (2010). Boruta – a system for feature selection. *Fundam. Inform.* 101 271–285. 10.3233/fi-2010-288

[B38] Lakso A. N. (1980). Aspects of canopy photosynthesis and productivity in the apple tree. *Acta Hortic.* 114 100–109.

[B39] Langmead B. Salzberg S. L. (2012). Fast gapped-read alignment with Bowtie 2. *Nat. Methods* 9 357–359. 10.1038/nmeth.1923 22388286 PMC3322381

[B40] Lawo S. Bashkurov M. Mullin M. Ferreria M. G. Kittler R. Habermann B. (2009). HAUS, the 8-subunit human Augmin complex, regulates centrosome and spindle integrity. *Curr. Biol.* 19 816–826. 10.1016/j.cub.2009.04.033 19427217

[B41] Layne R. E. C. Quamme H. A. (1975). “Pears”, in *Advances in Fruit Breeding*, eds Janick J. Moore J. N. (West Lafayette, IN: Purdue University Press), 38–70.

[B42] Li B. Dewey C. N. (2011). RSEM: accurate transcript quantification from RNA-Seq data with or without a reference genome. *BMC Bioinform.* 12:323. 10.1186/1471-2105-12-323 21816040 PMC3163565

[B43] Linsmith G. Rombauts S. Montanari S. Deng C. H. Celton J. M. Guerif P. (2019). Pseudo-chromosome-length genome assembly of a double haploid “Bartlett” pear (*Pyrus communis* L.). *Gigascience* 8:giz138. 10.1093/gigascience/giz138 31816089 PMC6901071

[B44] Liu T. Tian J. Wang G. Yu Y. Wang C. Ma Y. (2014). Augmin triggers microtubule-dependent microtubule nucleation in interphase plant cells. *Curr. Biol.* 24 2708–2713. 10.1016/j.cub.2014.09.053 25447999

[B45] Lobrich M. Jeggo P. A. (2007). The impact of a negligent G2/M checkpoint on genomic instability and cancer induction. *Nat. Rev. Cancer* 7 861–869. 10.1038/nrc2248 17943134

[B46] Love M. I. Huber W. Anders S. (2014). Moderated estimation of fold change and dispersion for RNA-seq data with DESeq2. *Genome Biol.* 15:550. 10.1186/s13059-014-0550-8 25516281 PMC4302049

[B47] Lu P. Yu S. Zhu N. Chen Y. R. Zhou B. Pan Y. (2018). Genome encode analyses reveal the basis of convergent evolution of fleshy fruit ripening. *Nat. Plants* 4 784–791. 10.1038/s41477-018-0249-z 30250279

[B48] Lurie S. Watkins C. B. (2012). Superficial scald, its etiology and control. *Postharvest Biol. Technol.* 65 44–60. 10.1016/j.postharvbio.2011.11.001

[B49] Mateo-Bonmatí E. Esteve-Bruna D. Juan-Vicente L. Nadi R. Candela H. Lozano F. M. (2018). INCURVATA11 and CUPULIFORMIS2 are redundant genes that encode epigenetic machinery components in *Arabidopsis*. *Plant Cell* 30 1596–1616. 29915151 10.1105/tpc.18.00300 PMC6096603

[B50] McGuire R. G. (1992). Reporting of objective color measurements. *HortScience* 27 1254–1255. 10.21273/hortsci.27.12.1254

[B51] Musacchi S. (2008). Training systems and soil management for Southern European pear orchards. *Acta Hortic.* 772 447–457. 10.17660/ActaHortic.2008.772.76

[B52] Noferini M. Fiori G. Costa G. (2006). *Method and Apparatus for Determining Quality of Fruit and Vegetable Products.* Dissertation. Bologna: Università di Bologna.

[B53] Nunez-Delicado E. Serrano-Megias M. Perez-Lopez A. J. Lopez-Nicolas J. M. (2005). Polyphenol oxidase from ‘Dominga’ table grape. *J. Agric. Food Chem.* 53 6087–6093. 10.1021/jf050346z 16029000

[B54] Palmer J. W. Giuliani R. Adams H. M. (1997). Effect of crop load on fruiting and leaf photosynthesis of ‘Braeburn’/M.26 apple trees. *Tree Physiol.* 17 741–746. 10.1093/treephys/17.11.741 14759899

[B55] Predieri S. Gatti E. Rapparini F. Cavicchi L. Colombo L. (2005). Sensory evaluation from a consumer perspective and its application to ‘Abbé Fétel’ pear fruit quality. *Acta Hortic.* 671 349–353.

[B56] Priness I. Maimon O. Ben-Gal I. (2007). Evaluation of gene-expression clustering via mutual information distance measure. *BMC Bioinform.* 8:111. 10.1186/1471-2105-8-111 17397530 PMC1858704

[B57] Qi F. Zhang F. (2019). Cell cycle regulation in the plant response to stress. *Front. Plant Sci.* 10:1765. 10.3389/fpls.2019.01765 32082337 PMC7002440

[B58] R Core Team (2019). *R: A Language and Environment for Statistical Computing.* Vienna: R Foundation for Statistical Computing.

[B59] Ramos D. E. Weinbaum S. A. Shackel K. A. Schwankl L. J. Mitcham E. J. Mitchell F. G. (1994). Influence of tree water status and canopy position on fruit size and quality of ‘Bartlett’ pears. *Acta Hortic.* 367 192–200. 10.17660/ActaHortic.1994.367.24

[B60] Roca J. (2009). Topoisomerase II: a fitted mechanism for the chromatin landscape. *Nucleic Acids Res.* 37 721–730. 10.1093/nar/gkn994 19059997 PMC2647320

[B61] Rudnizky S. Malik O. Bavly A. Pnueli L. Melamed P. Kaplan A. (2017). Nucleosome mobility and the regulation of gene expression: insights from single-molecule studies. *Protein Sci.* 26 1266–1277. 10.1002/pro.3159 28329910 PMC5477540

[B62] Saelens W. Cannoodt R. Saeys Y. (2018). A comprehensive evaluation of module detection methods for gene expression data. *Nat. Commun.* 9:1090. 10.1038/s41467-018-03424-4 29545622 PMC5854612

[B63] Serra S. Leisso R. Giordani L. Kalcsits L. Musacchi S. (2016). Crop load influences fruit quality, nutritional balance, and return bloom in ‘Honeycrisp’ apple. *HortScience* 51 236–244. 10.21273/hortsci.51.3.236

[B64] Serra S. Sullivan N. Mattheis J. P. Musacchi S. Rudell D. R. (2018). Canopy attachment position influences metabolism and peel constituency of European pear fruit. *BMC Plant Biol.* 18:364. 10.1186/s12870-018-1544-6 30563450 PMC6299602

[B65] Shealy B. T. Burns J. J. R. Smith M. C. Alex Feltus F. Ficklin S. P. (2019). GPU implementation of pairwise gaussian mixture models for multi-modal gene co-expression networks. *IEEE Access* 7 160845–160857. 10.1109/access.2019.2951284

[B66] Smoot M. E. Ono K. Ruscheinski J. Wang P. L. Ideker T. (2011). Cytoscape 2.8: new features for data integration and network visualization. *Bioinformatics* 27 431–432. 10.1093/bioinformatics/btq675 21149340 PMC3031041

[B67] Song L. Langfelder P. Horvath S. (2012). Comparison of co-expression measures: mutual information, correlation, and model based indices. *BMC Bioinform.* 13:328. 10.1186/1471-2105-13-328 23217028 PMC3586947

[B68] Stephan J. Sinoquet H. Dones N. Haddad N. Talhouk S. Lauri P. E. (2008). Light interception and partitioning between shoots in apple cultivars influenced by training. *Tree Physiol.* 28 331–342. 10.1093/treephys/28.3.331 18171657

[B69] Vanoli M. Buccheri M. (2012). Overview of the methods for assessing harvest maturity. *Stewart Postharvest Rev.* 8 1–11. 10.2212/spr.2012.1.4 25112557

[B70] Veluchamy A. Jegu T. Ariel F. Latrasse D. Mariappan K. G. Kim S. K. (2016). LHP1 regulates H3K27me3 spreading and shapes the three-dimensional conformation of the *Arabidopsis* genome. *PLoS One* 11:e0158936. 10.1371/journal.pone.0158936 27410265 PMC4943711

[B71] Vidoni S. Fiori G. Rocchi L. Spinelli F. Musacchi S. Costa G. (2015). Dafl: new innovative device to monitor fruit ripening in storage. *Acta Hortic.* 1094 549–554. 10.17660/ActaHortic.2015.1094.73

[B72] Wang Y. (2016). Storage temperature, controlled atmosphere, and 1-methylcyclopropene effects on α-farnesene, conjugated trienols, and peroxidation in relation with superficial scald, pithy brown core, and fruit quality of ‘d’Anjou’ pears during long-term storage. *J. Am. Soc. Hortic. Sci.* 141 177–185.

[B73] Wang Y. Xie X. Sugar D. (2015). Effects of harvest maturity, production year, storage temperature, and post-storage ethylene conditioning on ripening capacity of 1-mcp treated ‘d’Anjou’ pear. *Acta Hortic.* 1094 573–578.

[B74] Wang Z. Du H. Zhai R. Song L. Ma F. Xu L. (2017). Transcriptome analysis reveals candidate genes related to color fading of ‘Red Bartlett’ (*Pyrus communis* L.). *Front. Plant Sci.* 8:455. 10.3389/fpls.2017.00455 28408914 PMC5374147

[B75] Warnes G. Bolker B. Bonebakker L. Gentleman R. Huber W. Liaw A. (2020). *gplots: Various r Programming Tools for Plotting Data. R-Project. R Package Version 2, no. 4: 1.*

[B76] Warrington I. J. Stanley C. J. Tustin D. S. Hirst P. M. Cashmore W. M. (1996). Light transmission, yield distribution, and fruit quality in six tree canopy forms of ‘Granny Smith’ apple. *J. Tree Fruit Prod.* 1 27–54.

[B77] Whitaker B. D. Villalobos-Acuña M. Mitcham E. J. Mattheis J. P. (2009). Superficial scald susceptibility and α-farnesene metabolism in ‘Bartlett’ pears grown in California and Washington. *Postharvest Biol. Technol.* 53 43–50. 10.1016/j.postharvbio.2009.04.002

[B78] Wu J. Wang Y. Xu J. Korban S. S. Fei Z. Tao S. (2018). Diversification and independent domestication of Asian and European pears. *Genome Biol.* 19:77. 10.1186/s13059-018-1452-y 29890997 PMC5996476

[B79] Yip A. M. Horvath S. (2007). Gene network interconnectedness and the generalized topological overlap measure. *BMC Bioinform.* 8:22. 10.1186/1471-2105-8-22 17250769 PMC1797055

[B80] Zhang J. Serra S. Leisso S. R. Musacchi S. (2016). Effect of light microclimate on the quality of ‘d’Anjou’ pears in mature open-centre tree architecture. *Biosyst. Eng.* 141 1–11. 10.1016/j.biosystemseng.2015.11.002

[B81] Ziosi V. Noferini M. Fiori G. Tadiello A. Trainotti L. Casadoro G. (2008). A new index based on vis spectroscopy to characterize the progression of ripening in peach fruit. *Postharvest Biol. Technol.* 49 319–329. 10.1016/j.postharvbio.2008.01.017

