# Supplementary material for: Transcriptomics of Differential Ripening in ‘d’Anjou’ Pear (Pyrus communis L.)
Source: Front Plant Sci. 2021 Jun 16;12:609684. doi: 10.3389/fpls.2021.609684 (PMC8243007; doi:10.3389/fpls.2021.609684)

Supplementary Figure 1. **A Principal Components Analysis (PCA) of the peel transcriptome data showed sufficient structure to distinguish fruit through time and also by canopy position.** This analysis was done with a pilot gene expression matrix (GEM) - cleaned and trimmed RNA-Seq data were mapped to the Bartlett Genome V1.0 to create the GEM used for the PCA.

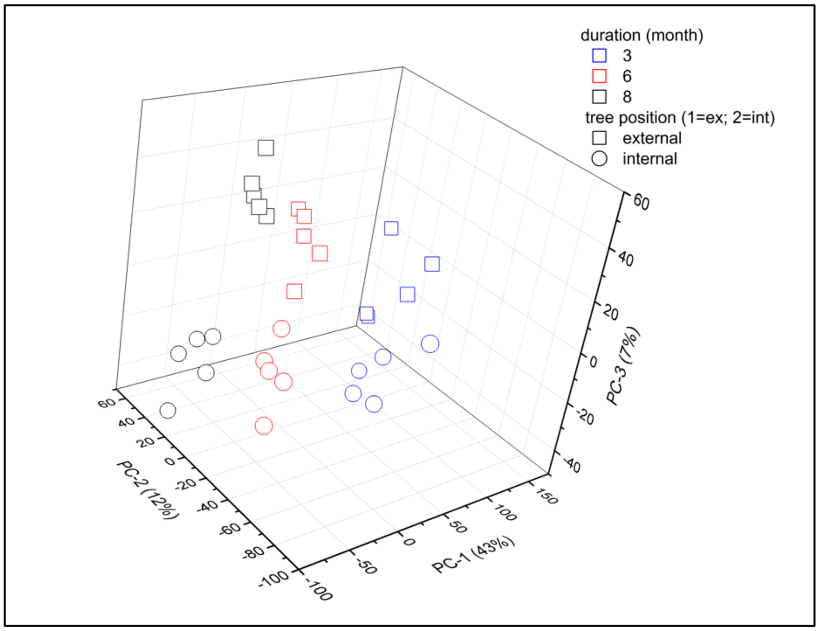

Supplement: Supplementary file 1 [file Image_1.PDF]
