# Supplementary material for: Transcriptomics of Differential Ripening in ‘d’Anjou’ Pear (Pyrus communis L.)
Source: Front Plant Sci. 2021 Jun 16;12:609684. doi: 10.3389/fpls.2021.609684 (PMC8243007; doi:10.3389/fpls.2021.609684)

Supplementary Figure 2. **There is substantial overlap among the thousands of genes that are differentially expressed at 3m postharvest.** VENN showing overlap of genes that are differentially expressed from harvest to 3 months of controlled atmosphere storage at -0.5˚C for both tissues and canopy positions. Significant differential expression was determined by Bonferroni $P<0.05$.

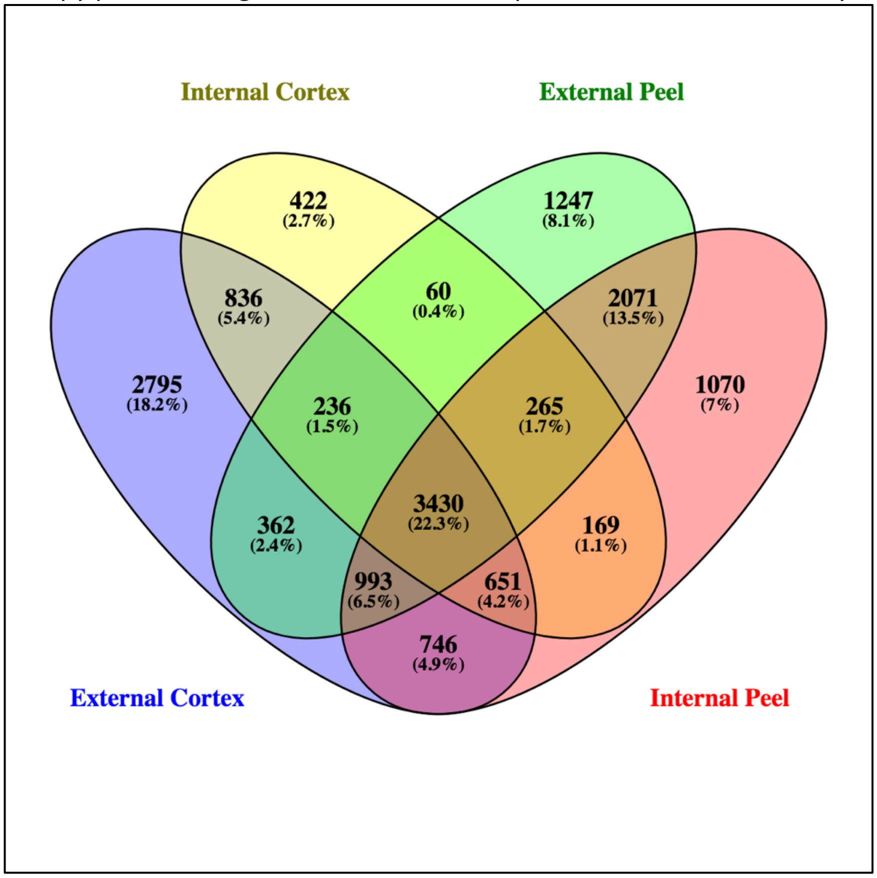

Supplement: Supplementary file 2 [file Image_2.pdf]
