# Supplementary material for: Transcriptomics of Differential Ripening in ‘d’Anjou’ Pear (Pyrus communis L.)
Source: Front Plant Sci. 2021 Jun 16;12:609684. doi: 10.3389/fpls.2021.609684 (PMC8243007; doi:10.3389/fpls.2021.609684)

Supplementary Figure 3. **Cortical differential expression from internal fruit tended to be subset of external fruit, while peel differential expression was proportional with substantial overlap.** This was observed at all postharvest timepoints. Significant differential expression was determined by Bonferroni corrected P<0.05.

A. Number of DEGs 3m vs 6m Cortex

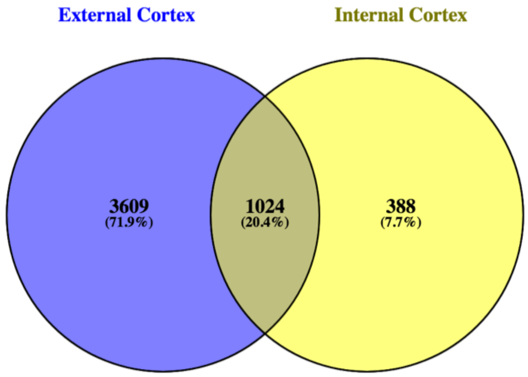

B. Number of DEGs 6m vs 8m Cortex

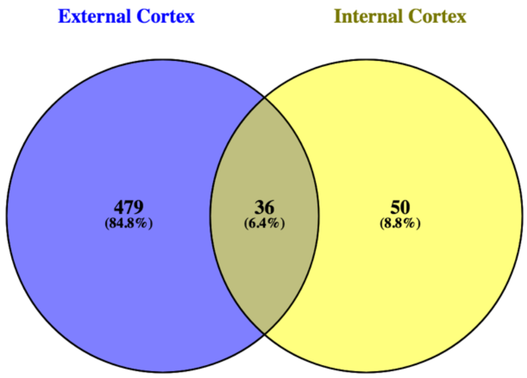

C. Number of DEGs 3m vs 6m Peel

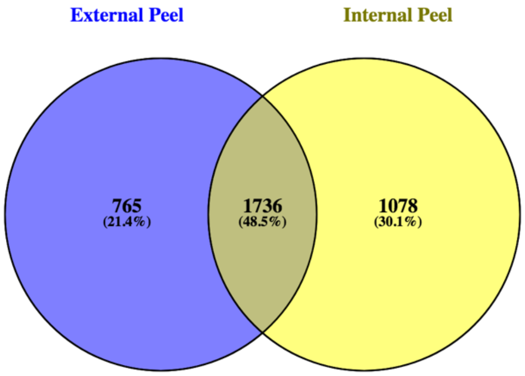

D. Number of DEGs 6m vs 8m Peel

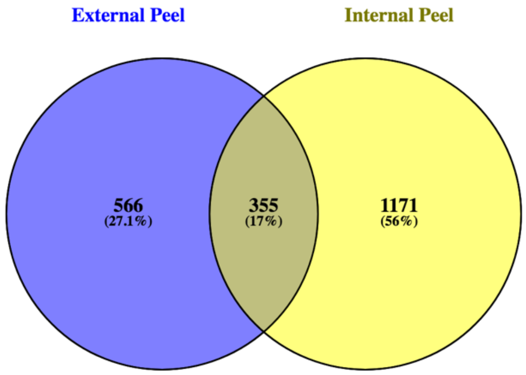

Supplement: Supplementary file 3 [file Image_3.PDF]
