# Supplementary material for: Transcriptomics of Differential Ripening in ‘d’Anjou’ Pear (Pyrus communis L.)
Source: Front Plant Sci. 2021 Jun 16;12:609684. doi: 10.3389/fpls.2021.609684 (PMC8243007; doi:10.3389/fpls.2021.609684)

Supplementary Figure 4. **Independent Z-score scaling for internal canopy position samples emphasizes phased character of expression based on canopy position (i.e. maturity).** See Figure 7A for comparison.

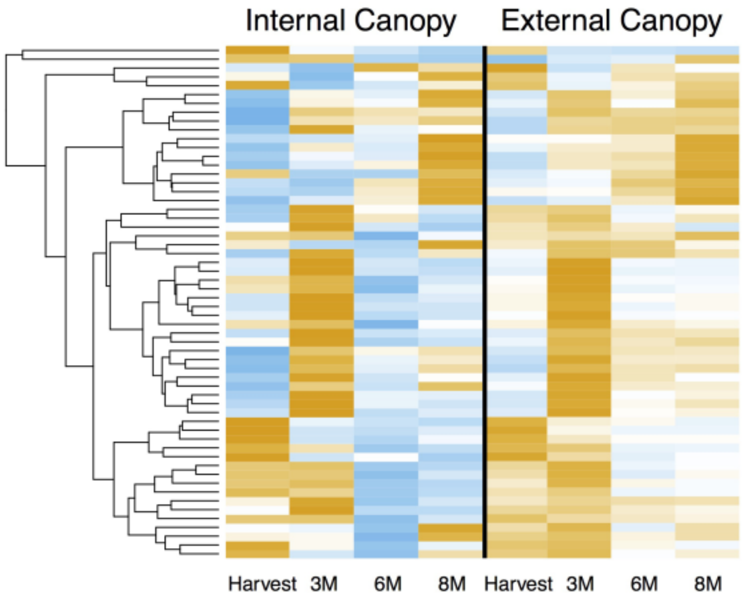

Supplement: Supplementary file 4 [file Image_4.PDF]
