# Supplementary material for: Transcriptomics of Differential Ripening in ‘d’Anjou’ Pear (Pyrus communis L.)
Source: Front Plant Sci. 2021 Jun 16;12:609684. doi: 10.3389/fpls.2021.609684 (PMC8243007; doi:10.3389/fpls.2021.609684)
Supplement: Supplementary file 5 [file Table_1.DOCX]

Supplementary Table 1. **RNA-Seq validation with qPCR shows agreement for candidate genes.** Gene IDs for best BLASTn hits are listed for both versions of the ‘Bartlett’ genome, as well as *de novo* assembled transcripts from our ‘d’Anjou’ data. N % ID = nucleotide percent identity and AA % ID = amino acid percent identity between the indicated sequences. R^2^ = Pearson’s r of biological replicates, not means.

| Gene Sequence IDs | | | Bartlett V1.0 RNA-Seq vs d’Anjou qPCR | | | Bartlett V1.0 RNA-Seq vs BartlettDH V2.0 RNA-Seq | | | BartlettDH V2.0 RNA-Seq vs d’Anjou qPCR | | |
| --- | --- | --- | --- | --- | --- | --- | --- | --- | --- | --- | --- |
| Bartlett V1.0 | BartlettDH  V2.0 | *de novo* D'Anjou | N  % ID | AA  % ID | R^2^ | N  % ID | AA  % ID | R^2^ | N  % ID | AA  % ID | R^2^ |
| PCP000169 | pycom111g04030 | contig_2870 | 97.5 | 96.7 | 0.96 | 99.6 | 100.0 | 0.49 | 88.4 | 96.7 | 0.60 |
| PCP002552 | pycom05g02020 | contig_1317 | 100.0 | 100.0 | 0.29 | 83.7 | 80.2 | 0.67 | 83.7 | 80.2 | 0.03 |
| PCP012051 | pycom02g14370 | contig_669 | 99.3 | 99.3 | 0.74 | 100.0 | 100.0 | 0.93 | 99.3 | 99.3 | 0.73 |
| PCP019300 | pycom15g13950 | contig_1489 | 100.0 | 100.0 | 0.84 | 100.0 | 100.0 | 0.94 | 100.0 | 100.0 | 0.82 |
| PCP019318 | pycom15g13690 | contig_1356 | 100.0 | 100.0 | 0.32 | 100.0 | 100.0 | 0.47 | 100.0 | 100.0 | 0.38 |
| PCP020138 | pycom03g07610 | contig_16103 | 92.1 | 93.3 | 0.98 | 100.0 | 100.0 | 0.93 | 100.0 | 100.0 | 0.92 |
| PCP022787 | pycom04g16930 | contig_2831 | 100.0 | 100.0 | 0.75 | 90.3 | 92.7 | 0.96 | 88.1 | 88.3 | 0.85 |
| PCP028936 | pycom17g10150 | contig_384 | 100.0 | 100.0 | 0.58 | 99.8 | 100.0 | 0.81 | 98.9 | 98.3 | 0.53 |
| PCP029268 | pycom01g10110 | contig_1630 | 99.3 | 99.3 | 0.85 | 100.0 | 100.0 | 0.95 | 99.3 | 99.3 | 0.85 |
| PCP045133 | pycom07g13220 | contig_71 | 99.4 | 99.5 | 0.87 | 97.9 | 97.4 | 0.89 | 98.7 | 99.1 | 0.69 |
