# Supplementary material for: Transcriptomics of Differential Ripening in ‘d’Anjou’ Pear (Pyrus communis L.)
Source: Front Plant Sci. 2021 Jun 16;12:609684. doi: 10.3389/fpls.2021.609684 (PMC8243007; doi:10.3389/fpls.2021.609684)
Supplement: Supplementary file 6 [file Table_2.DOCX]

Supplementary Table 2. **Subset of RNA samples used for qPCR validation of RNA-Seq data.**

| Time Point | Canopy Position | Tissue Type | Bio Rep ID |
| --- | --- | --- | --- |
| T0 | External | Cortex | Rep02 |
| T0 | External | Cortex | Rep03 |
| T0 | External | Cortex | Rep05 |
| T0 | External | Peel | Rep03 |
| T0 | External | Peel | Rep04 |
| T0 | External | Peel | Rep05 |
| T0 | Internal | Cortex | Rep02 |
| T0 | Internal | Cortex | Rep04 |
| T0 | Internal | Cortex | Rep05 |
| T0 | Internal | Peel | Rep01 |
| T0 | Internal | Peel | Rep02 |
| T0 | Internal | Peel | Rep04 |
| T2 | External | Peel | Rep02 |
| T2 | External | Peel | Rep03 |
| T2 | External | Peel | Rep04 |
| T2 | Internal | Cortex | Rep02 |
| T2 | Internal | Cortex | Rep03 |
| T2 | Internal | Cortex | Rep04 |
| T2 | Internal | Peel | Rep01 |
| T2 | Internal | Peel | Rep03 |
| T2 | Internal | Peel | Rep04 |
