# Supplementary material for: Transcriptomics of Differential Ripening in ‘d’Anjou’ Pear (Pyrus communis L.)
Source: Front Plant Sci. 2021 Jun 16;12:609684. doi: 10.3389/fpls.2021.609684 (PMC8243007; doi:10.3389/fpls.2021.609684)
Supplement: Supplementary file 7 [file Table_3.DOCX]

Supplementary Table 3. **Primers used for qPCR validation.**

| Bartlett V2.0 Gene ID | Forward Primer | Reverse Primer |
| --- | --- | --- |
| pycom111g04030 | GGAAGGTCAAGGCAGTCAGG | GCGCCTATCTCGAATCTCAGG |
| pycom05g02020 | GAAGACAAGCCCTCAGACTACC | CCAGAATAATCGTTGGTGTCGC |
| pycom02g14370 | AGGAAGTTGCGGGAGTTGC | CCGAGAGAGACACCAATTCTCC |
| pycom15g13950 | TGTCACGGGGGATTACTGG | CTGCCTGCTCAATATCCAAAGG |
| pycom15g13690 | GCATCTAAGACCTATCCCCAGC | GTCGAGACTTCAACAACCTTGC |
| pycom03g07610 | GCAAGTGTGGCATGTACCC | CCATAGCTCATCTCAGACCCC |
| pycom04g16930 | ATCACCTTCAAGACCCCTGC | CTTCCCCTTCCCATCCTTCC |
| pycom17g10150 | GGTAAGGGAGGAAAGAACAGG | AAGCACTTGGGCATACTCC |
| pycom01g10110 | CCTACAGCCATTCCATCCCC | CTTTAGTGATCCAGGGTCCGG |
| pycom07g13220 | CAAGCAAGAGATGTTCCAACCC | CCCCAGGAGTTATCAAGCCC |
