# Supplementary material for: Transcriptomics of Differential Ripening in ‘d’Anjou’ Pear (Pyrus communis L.)
Source: Front Plant Sci. 2021 Jun 16;12:609684. doi: 10.3389/fpls.2021.609684 (PMC8243007; doi:10.3389/fpls.2021.609684)
Supplement: Supplementary file 8 [file Table_4.DOCX]

Supplementary Table 4. **Pairwise comparisons for differential expression analysis for both peel and cortex.**

| Control Group | Test Group |
| --- | --- |
| Harvest (T0) Internal | T0 External |
|  | T1 Internal |
|  | T2 Internal |
|  | T3 Internal |
| Harvest (T0) External | T1 External |
|  | T2 External |
|  | T3 External |
| 3m CA (T1) |  |
| Internal | T1 External |
|  | T2 Internal |
| 3m CA (T1) |  |
| External | T2 External |
| 6m CA (T2) Internal | T2 External |
|  | T3 Internal |
| 6m CA (T2) External | T3 External |
| 8m CA (T3) Internal | T3 External |
