# Supplementary figures and images for: Transcriptomics of Differential Ripening in ‘d’Anjou’ Pear (Pyrus communis L.)
Source: Front Plant Sci. 2021 Jun 16;12:609684. doi: 10.3389/fpls.2021.609684 (PMC8243007; doi:10.3389/fpls.2021.609684)

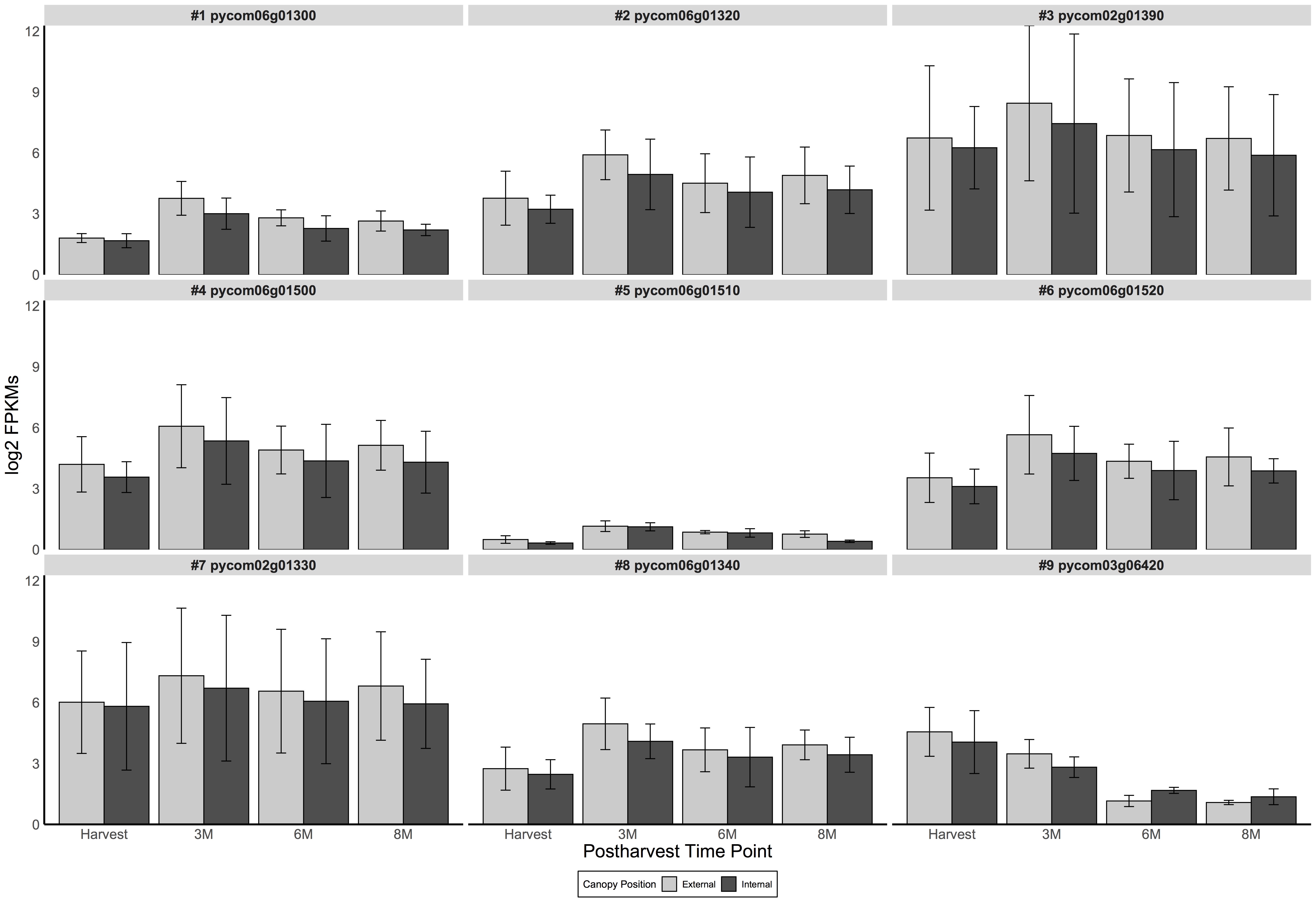

Supplement: Supplementary file 12 [file Data_Sheet_4.ZIP › Supp.4/Cortex AAT2.jpg]

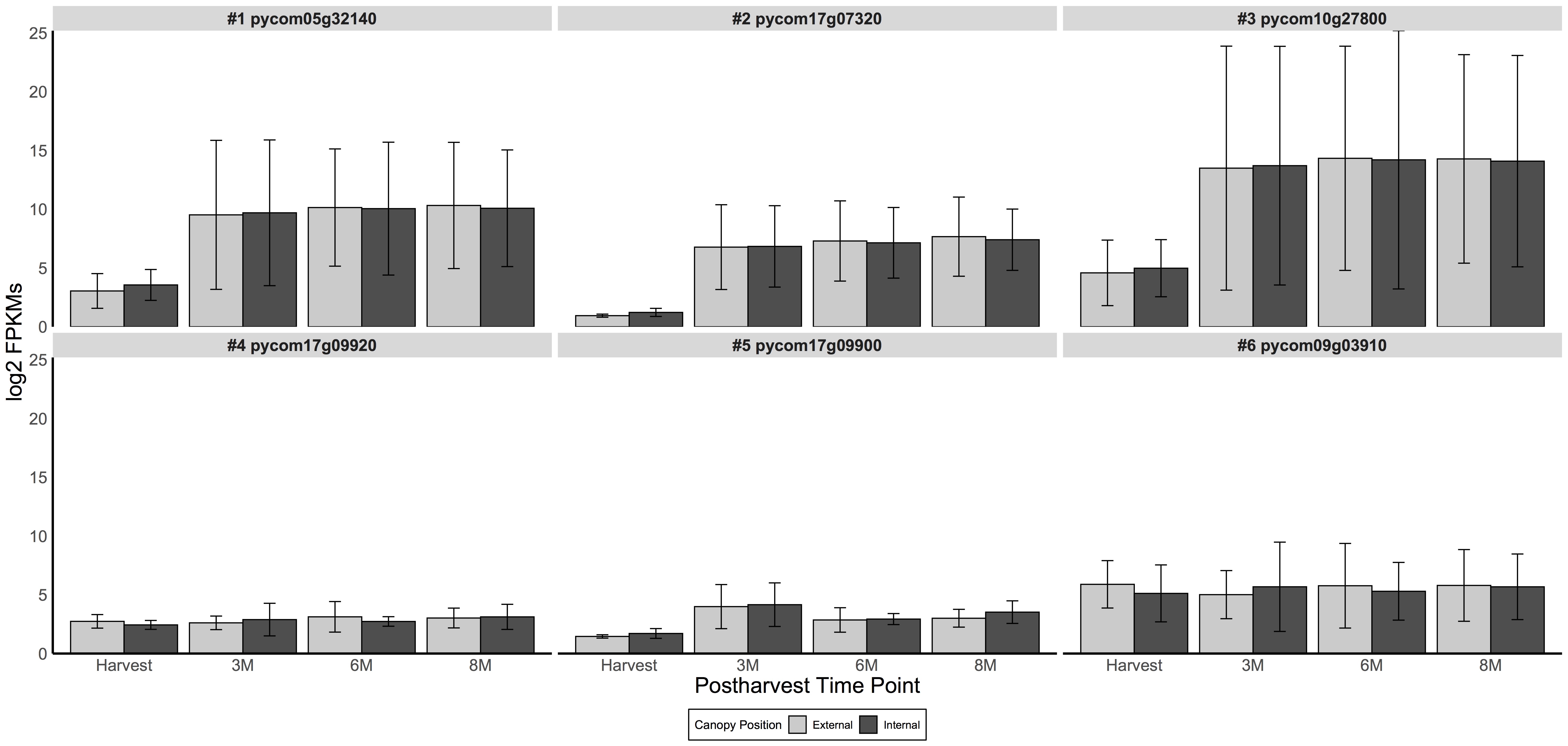

Supplement: Supplementary file 12 [file Data_Sheet_4.ZIP › Supp.4/Cortex ACO.jpg]

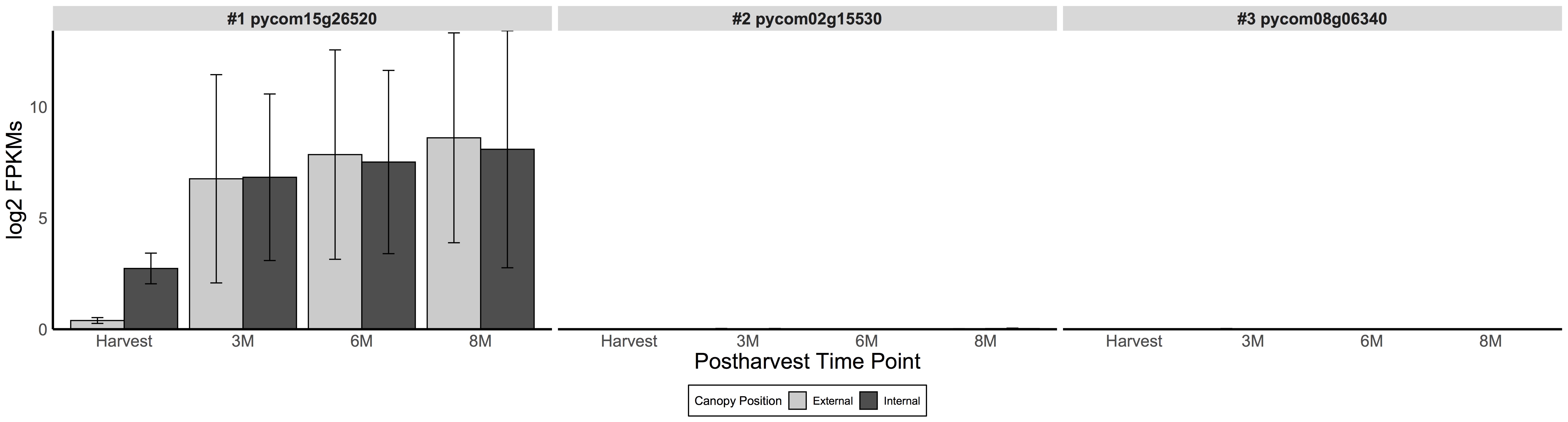

Supplement: Supplementary file 12 [file Data_Sheet_4.ZIP › Supp.4/Cortex ACS.jpg]

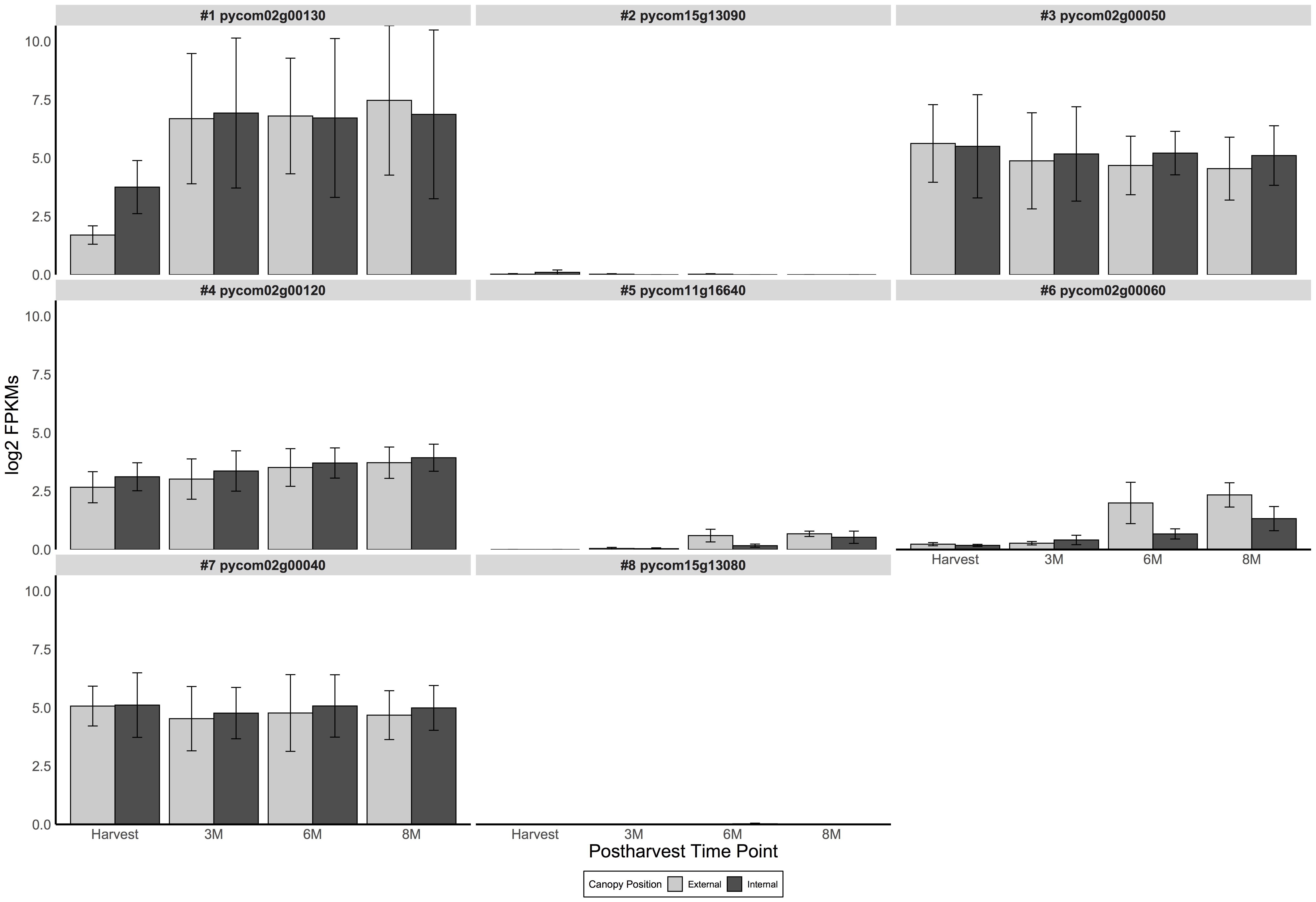

Supplement: Supplementary file 12 [file Data_Sheet_4.ZIP › Supp.4/Cortex ADH2.jpg]

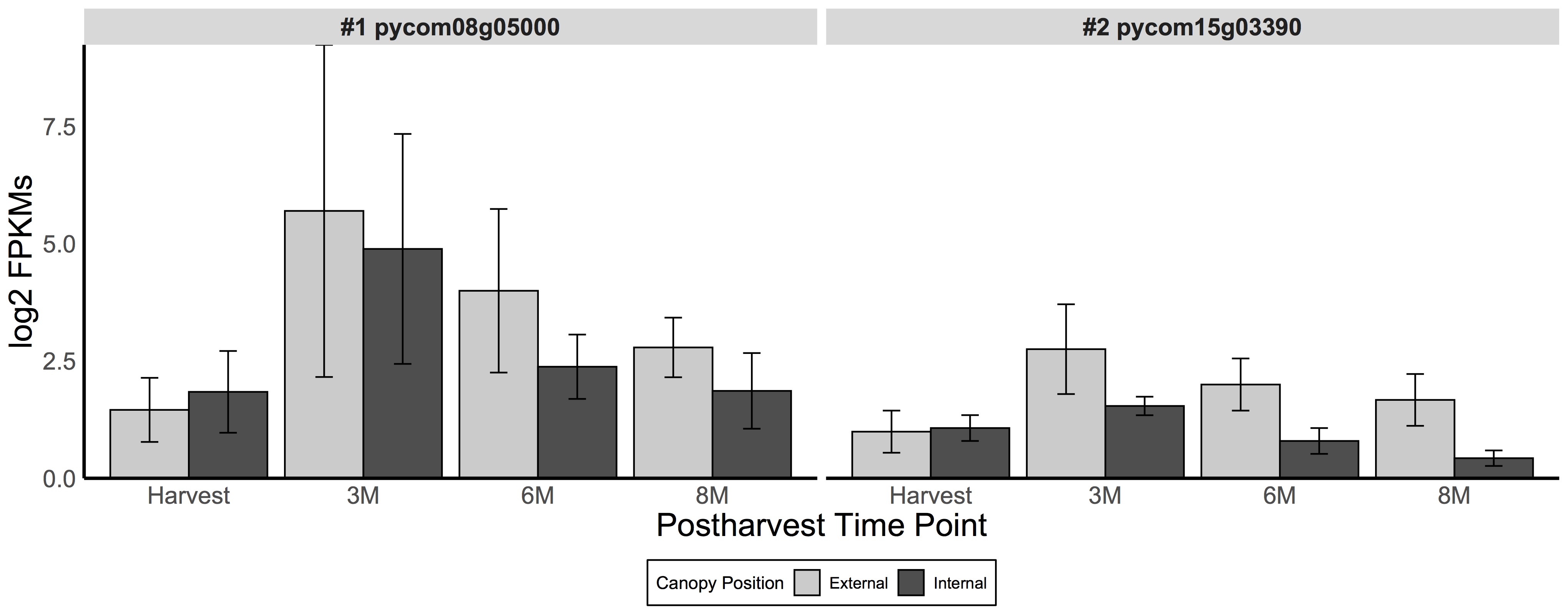

Supplement: Supplementary file 12 [file Data_Sheet_4.ZIP › Supp.4/Cortex ERF1.jpg]

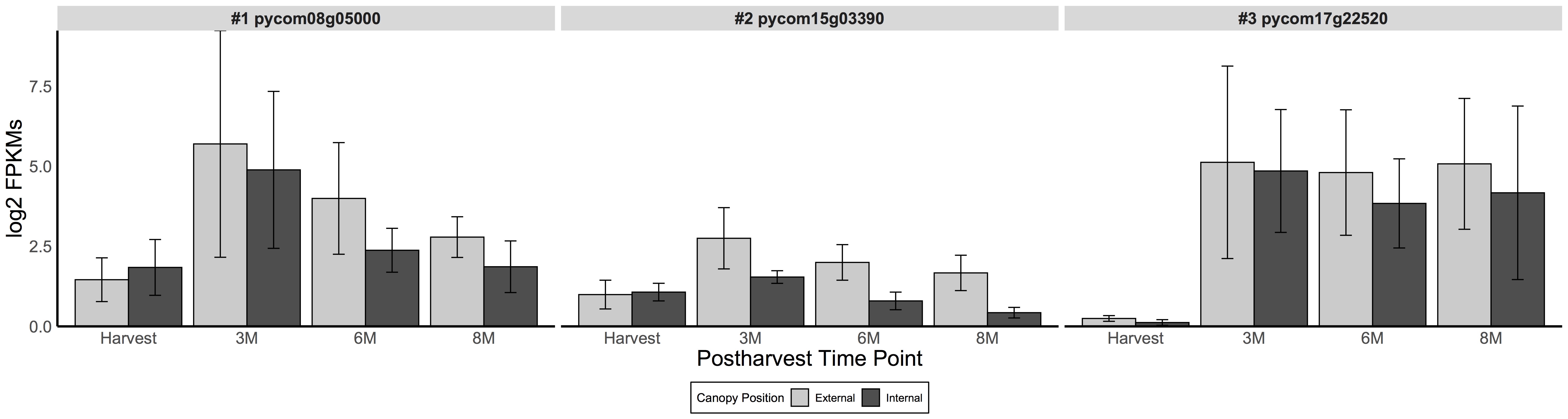

Supplement: Supplementary file 12 [file Data_Sheet_4.ZIP › Supp.4/Cortex ERF2.jpg]

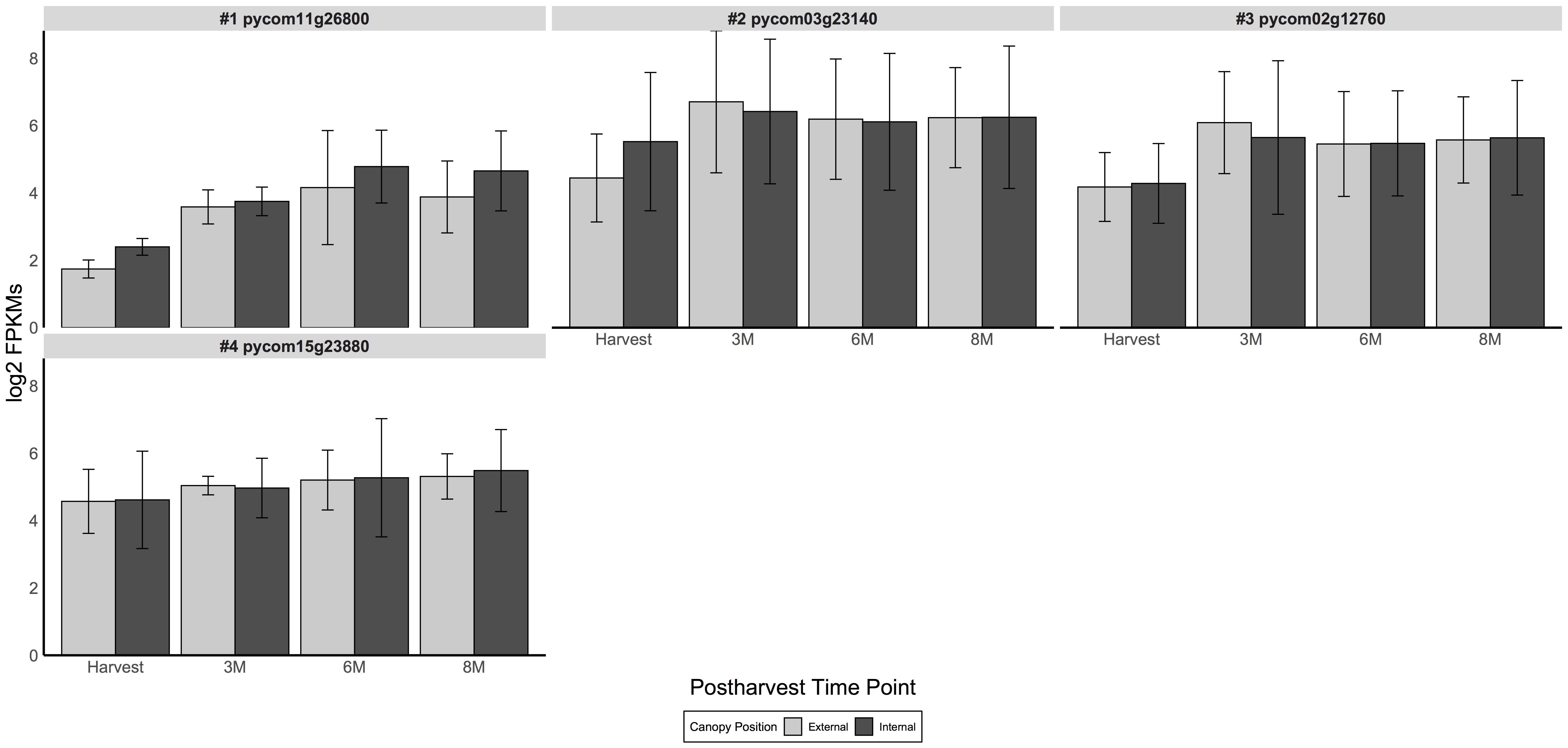

Supplement: Supplementary file 12 [file Data_Sheet_4.ZIP › Supp.4/Cortex ERS1.jpg]

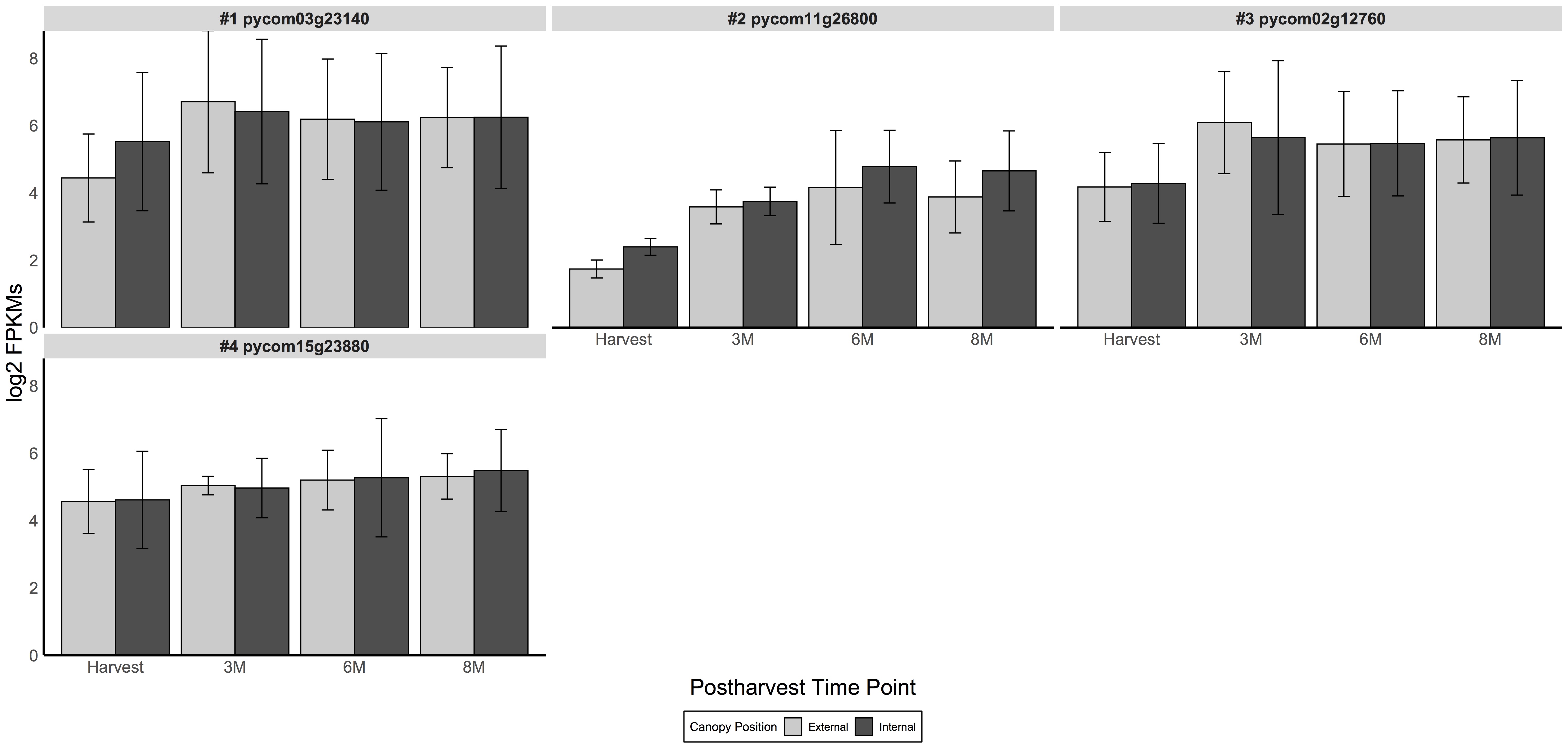

Supplement: Supplementary file 12 [file Data_Sheet_4.ZIP › Supp.4/Cortex ERS2.jpg]

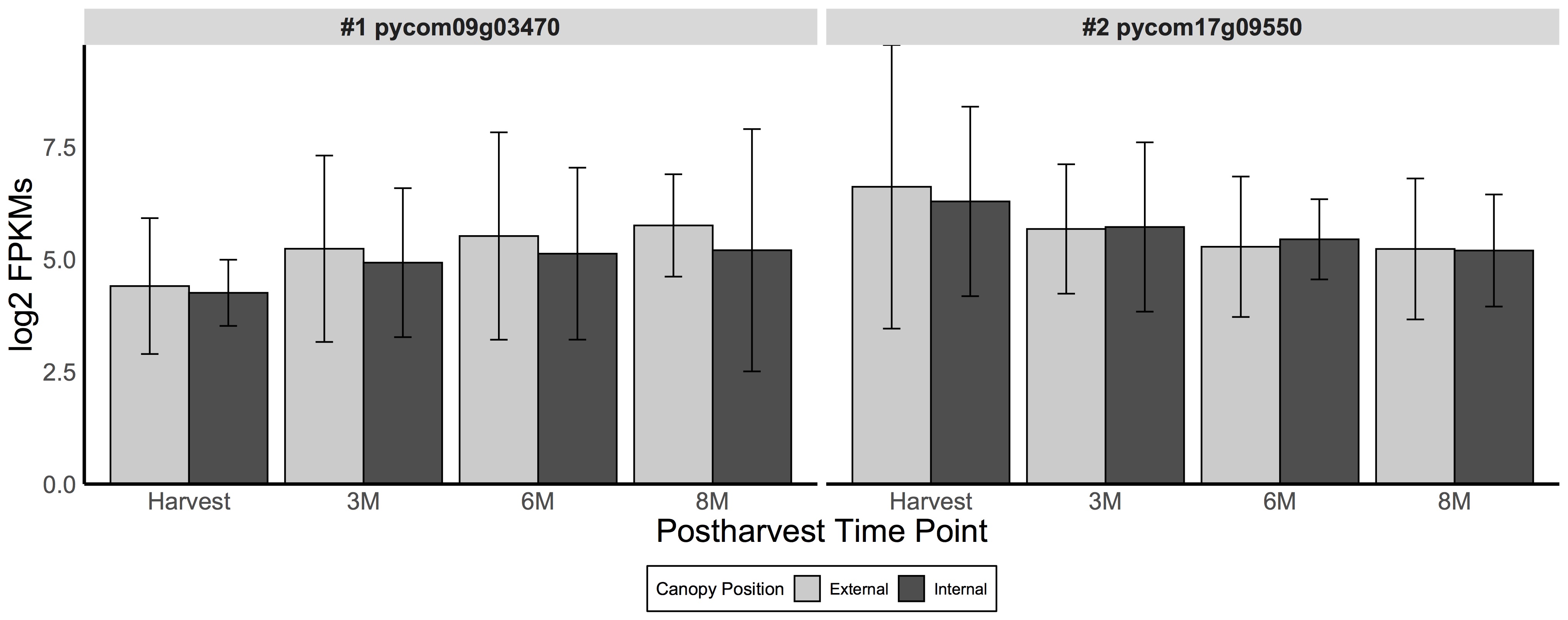

Supplement: Supplementary file 12 [file Data_Sheet_4.ZIP › Supp.4/Cortex HPL2.jpg]

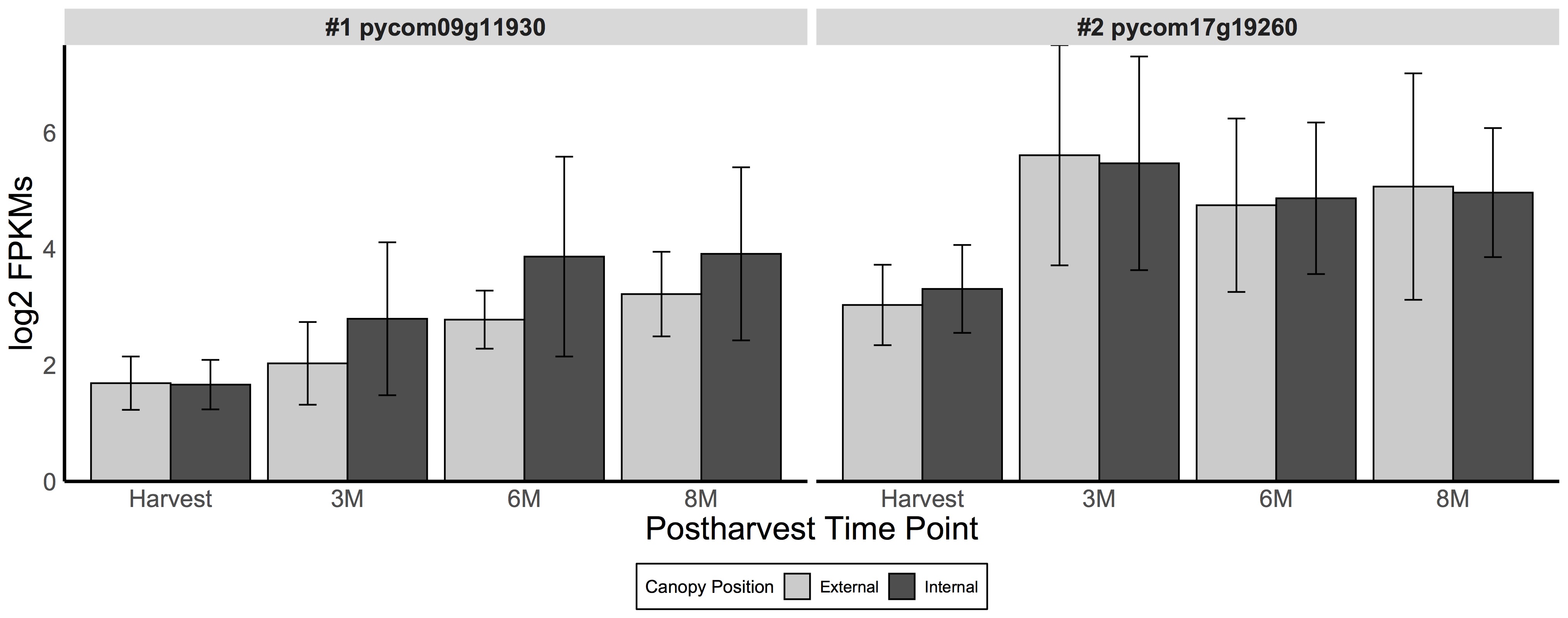

Supplement: Supplementary file 12 [file Data_Sheet_4.ZIP › Supp.4/Cortex IAA Protein.jpg]

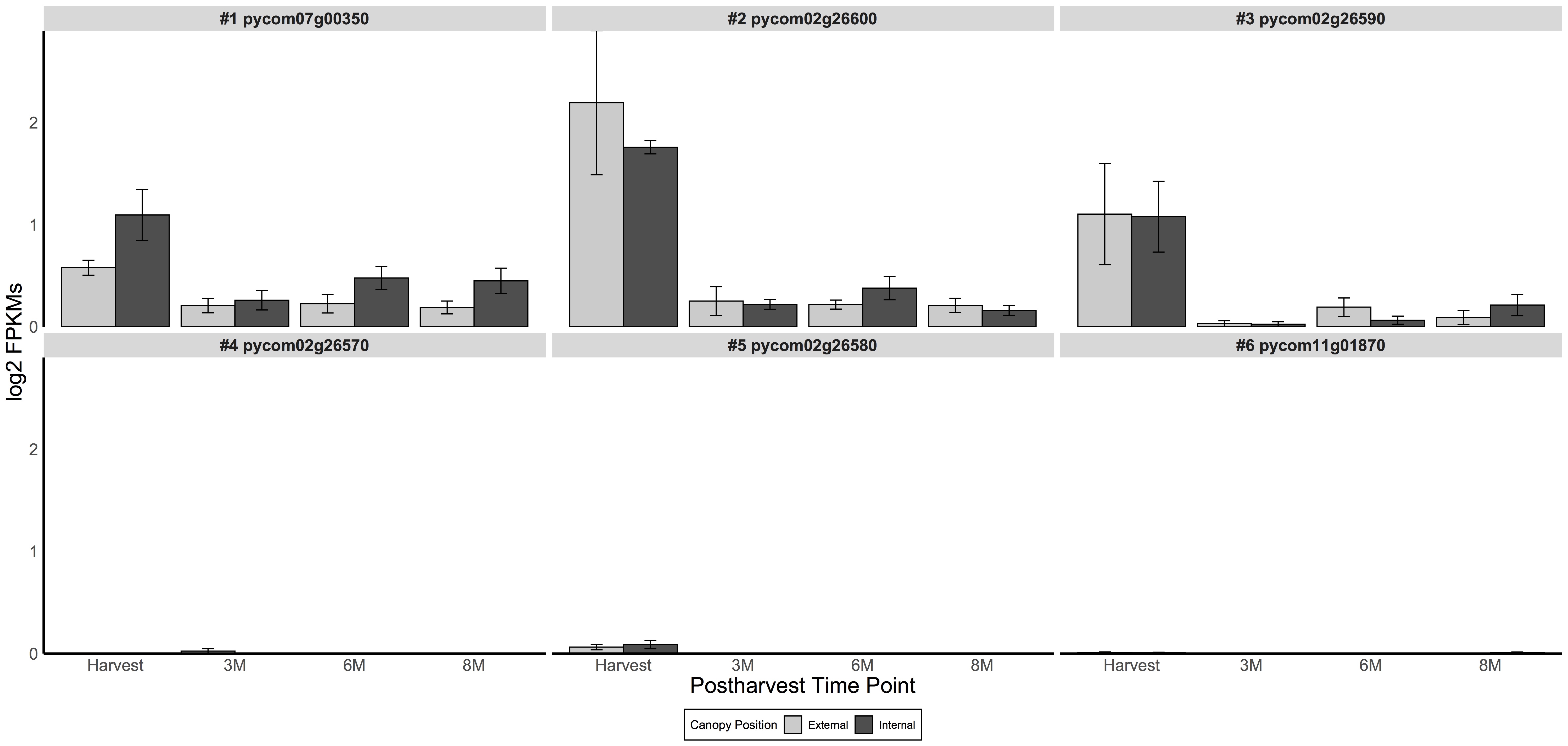

Supplement: Supplementary file 12 [file Data_Sheet_4.ZIP › Supp.4/Cortex LOX.jpg]

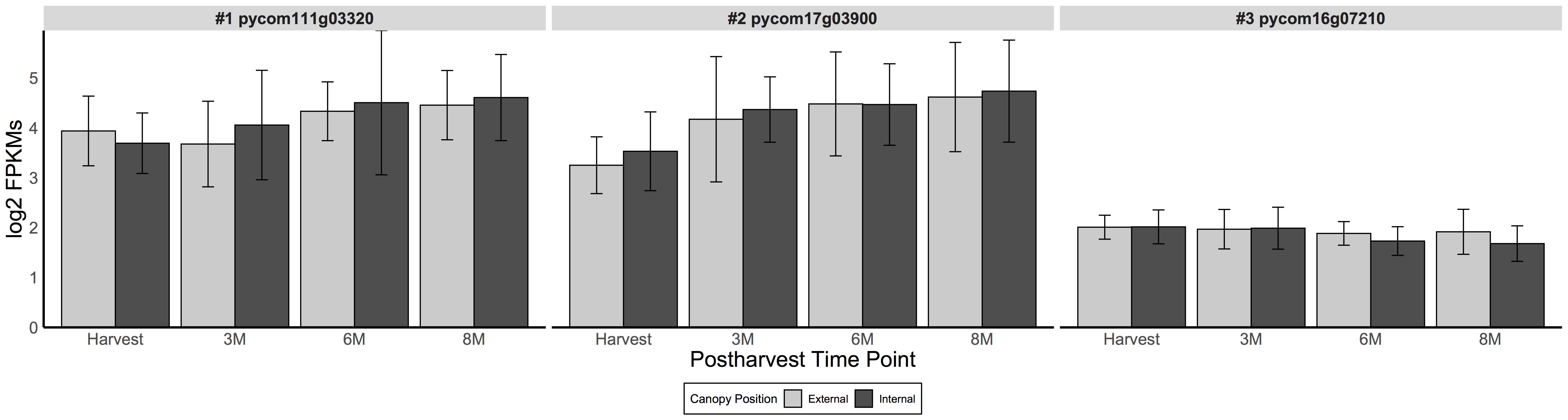

Supplement: Supplementary file 12 [file Data_Sheet_4.ZIP › Supp.4/Cortex MdGene-Housekeeping.jpg]

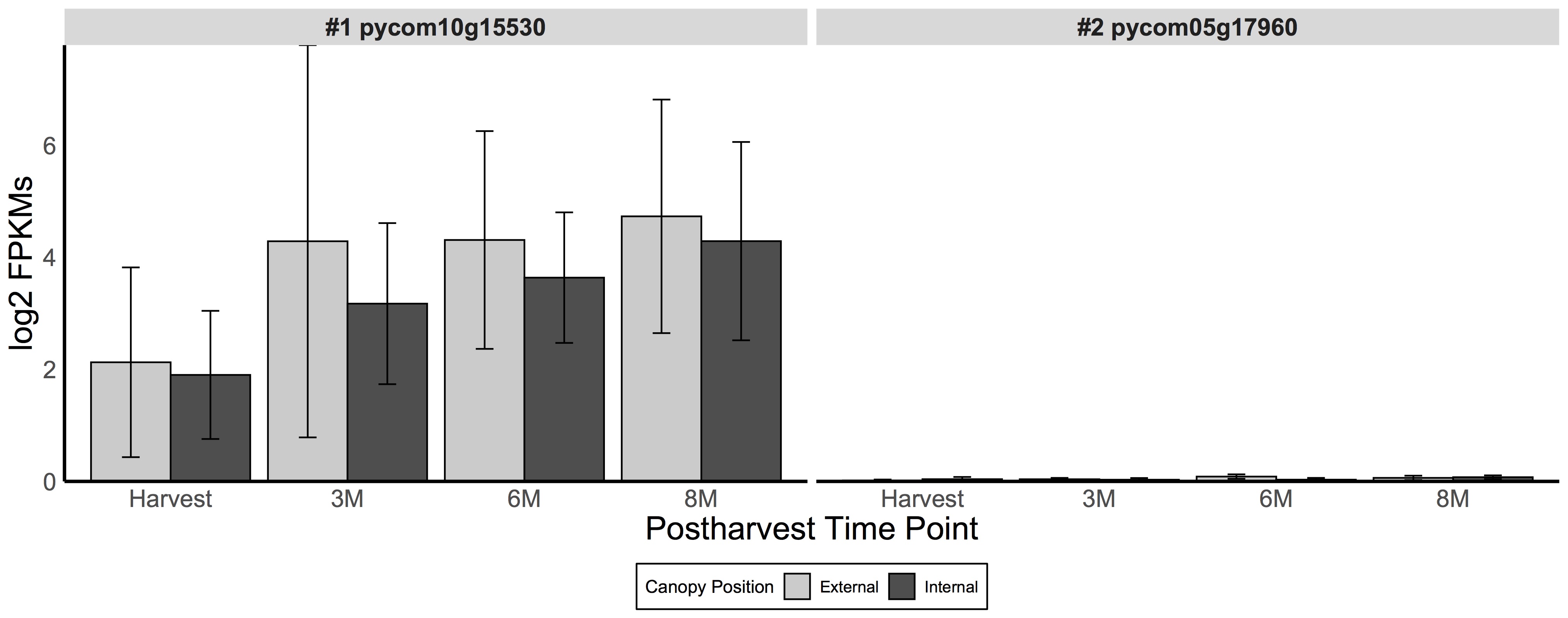

Supplement: Supplementary file 12 [file Data_Sheet_4.ZIP › Supp.4/Cortex PG.jpg]

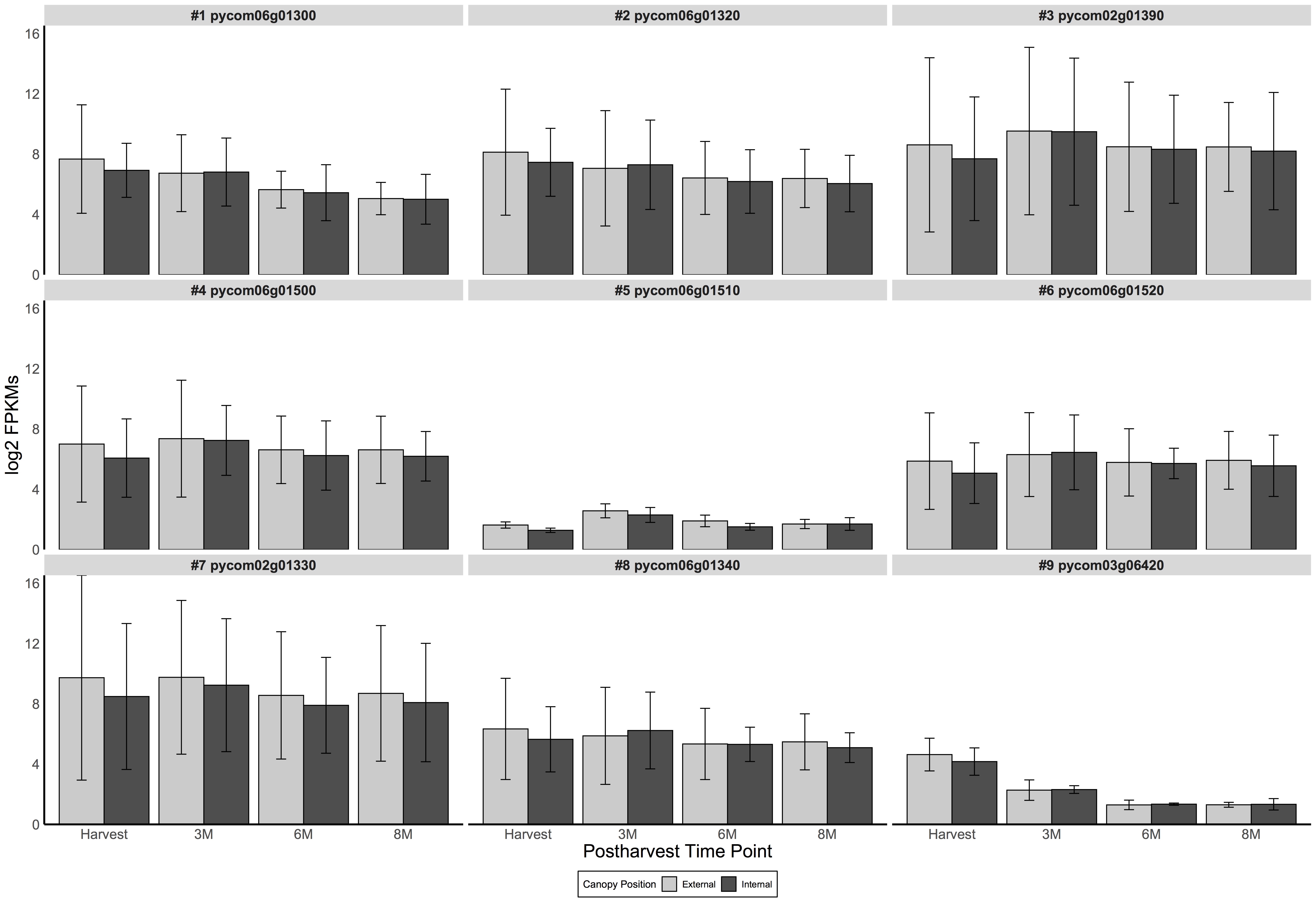

Supplement: Supplementary file 12 [file Data_Sheet_4.ZIP › Supp.4/Peel AAT2.jpg]

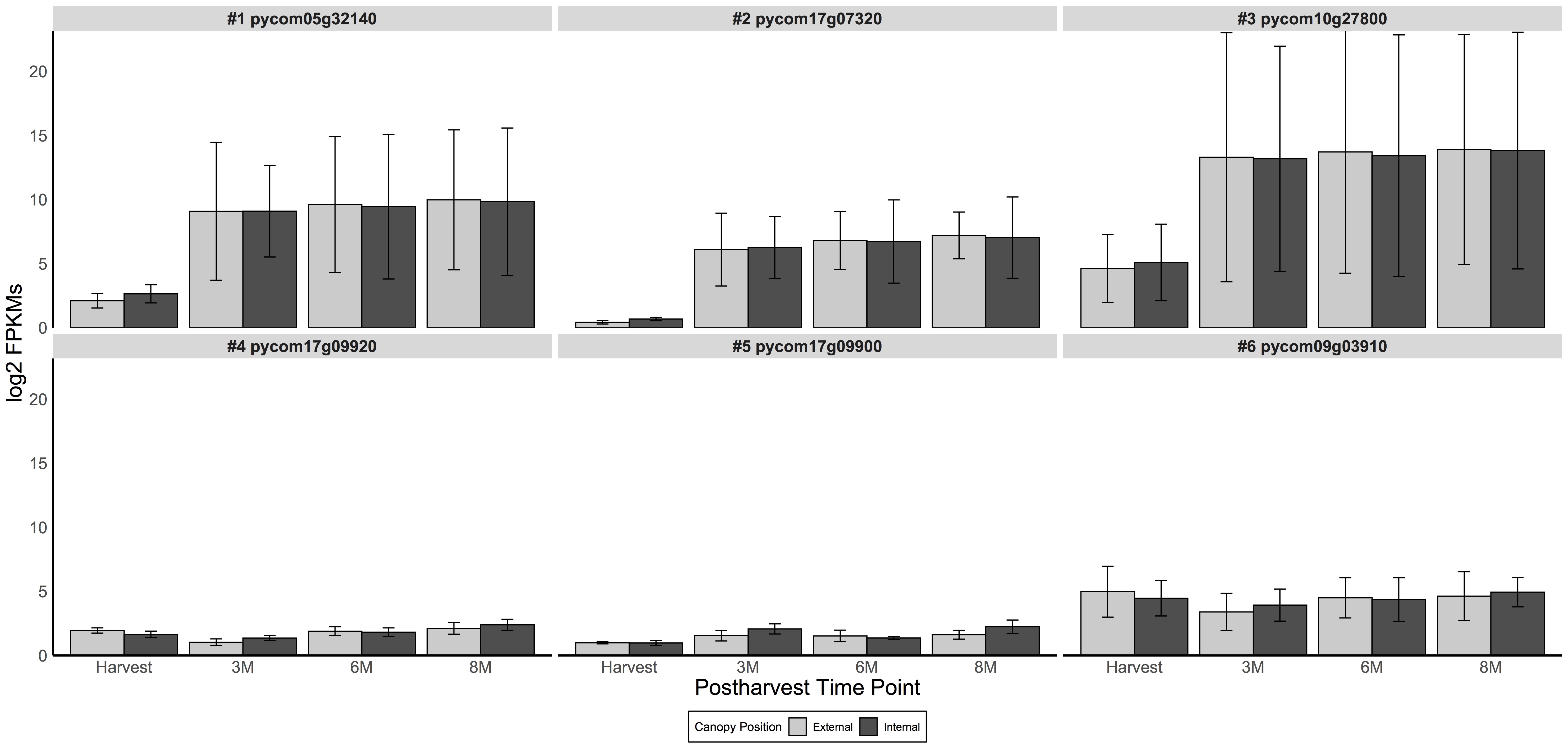

Supplement: Supplementary file 12 [file Data_Sheet_4.ZIP › Supp.4/Peel ACO.jpg]

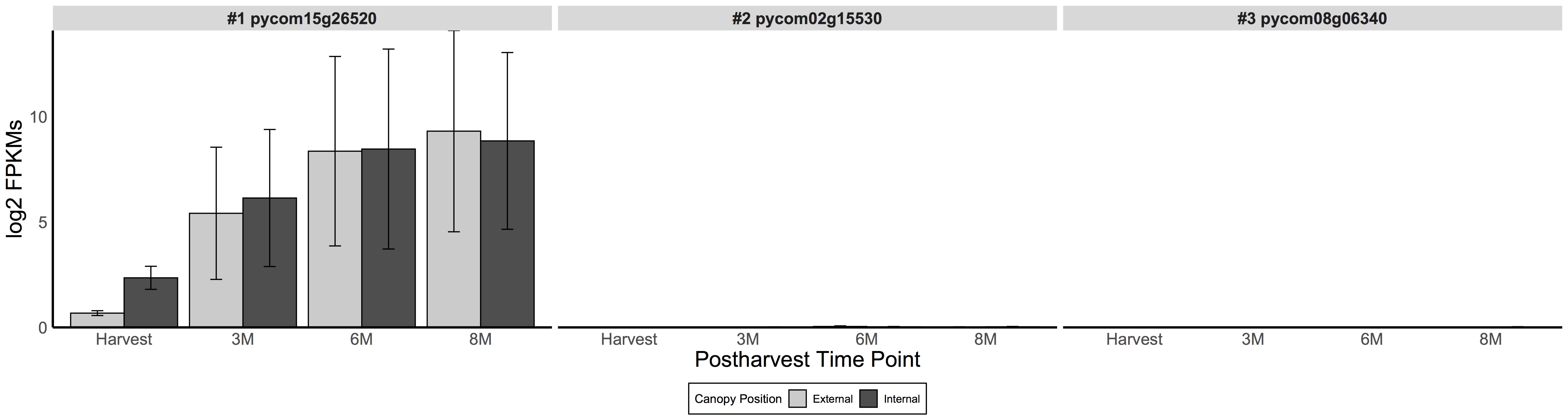

Supplement: Supplementary file 12 [file Data_Sheet_4.ZIP › Supp.4/Peel ACS.jpg]

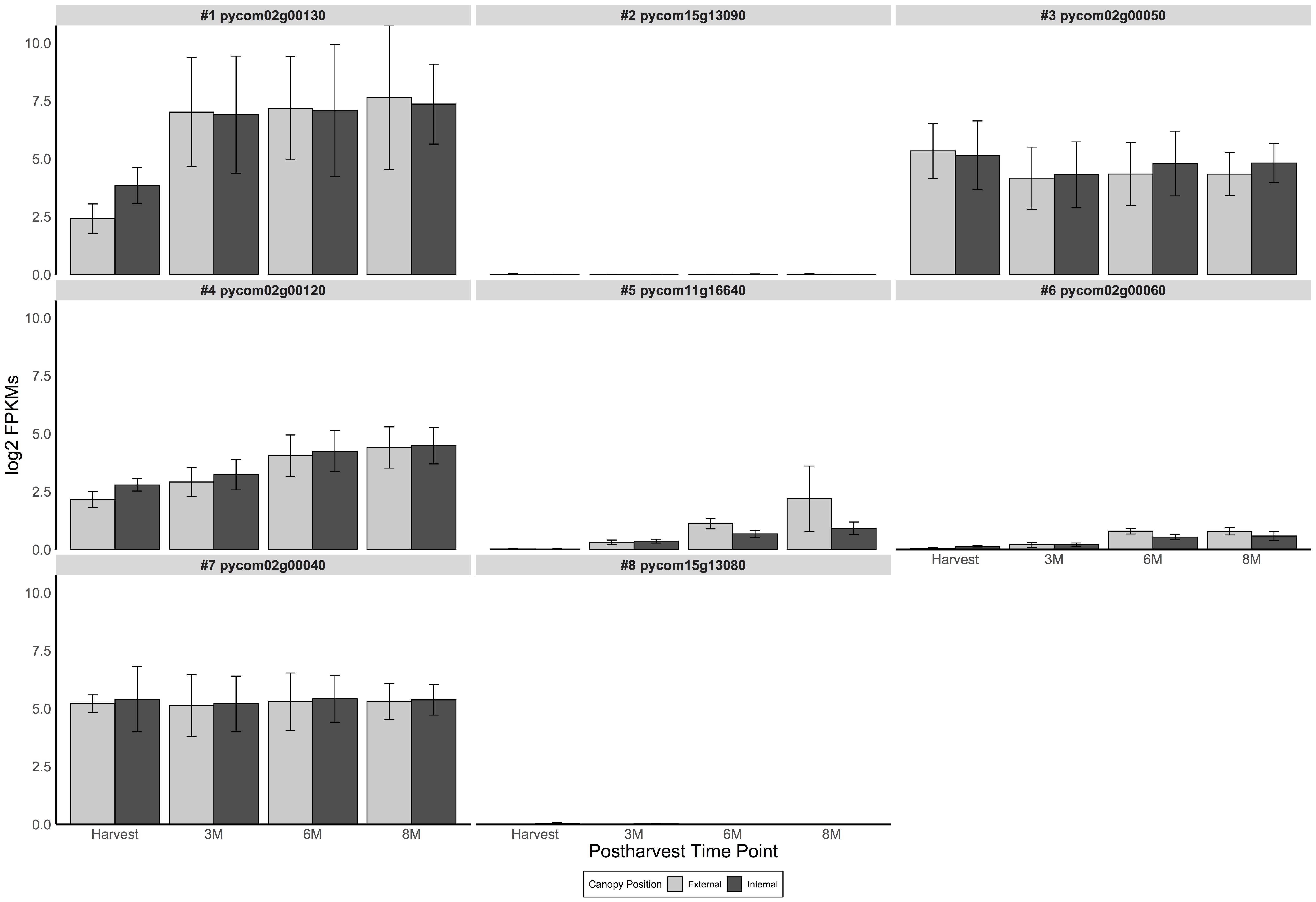

Supplement: Supplementary file 12 [file Data_Sheet_4.ZIP › Supp.4/Peel ADH2.jpg]

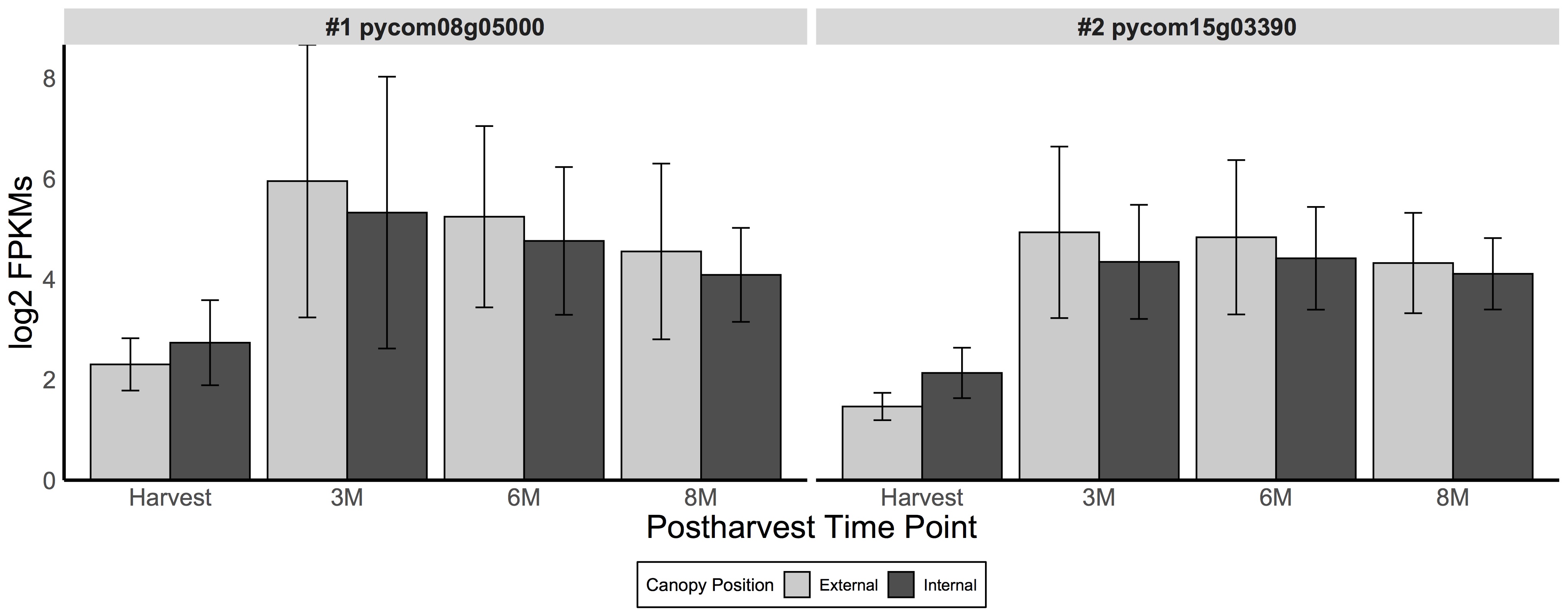

Supplement: Supplementary file 12 [file Data_Sheet_4.ZIP › Supp.4/Peel ERF1.jpg]

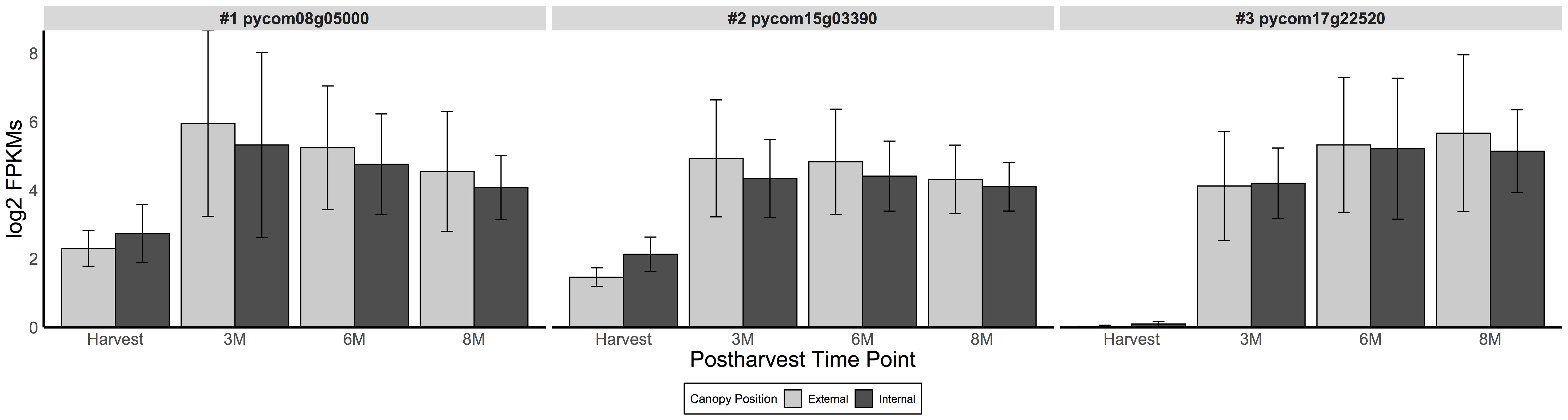

Supplement: Supplementary file 12 [file Data_Sheet_4.ZIP › Supp.4/Peel ERF2.jpg]

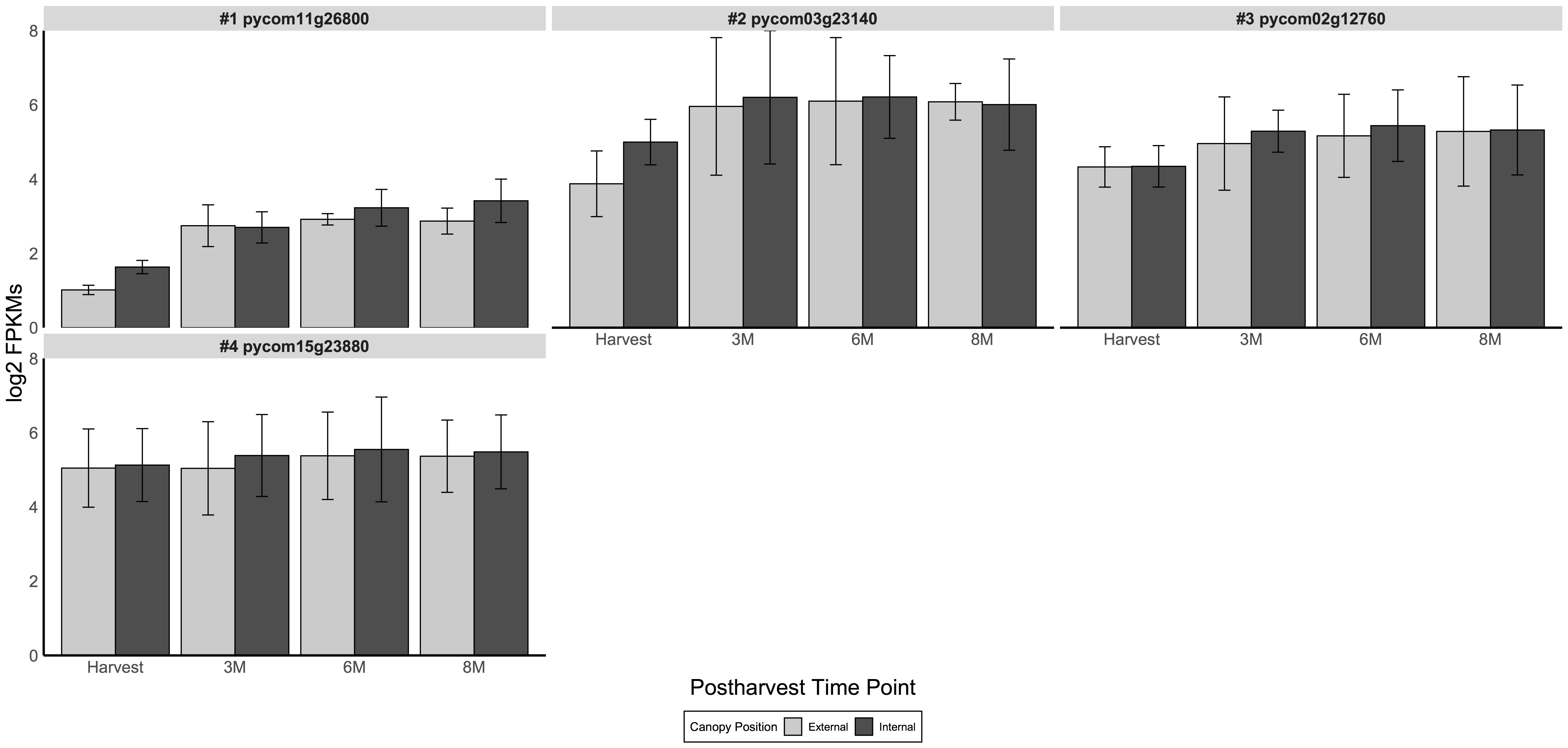

Supplement: Supplementary file 12 [file Data_Sheet_4.ZIP › Supp.4/Peel ERS1.jpg]

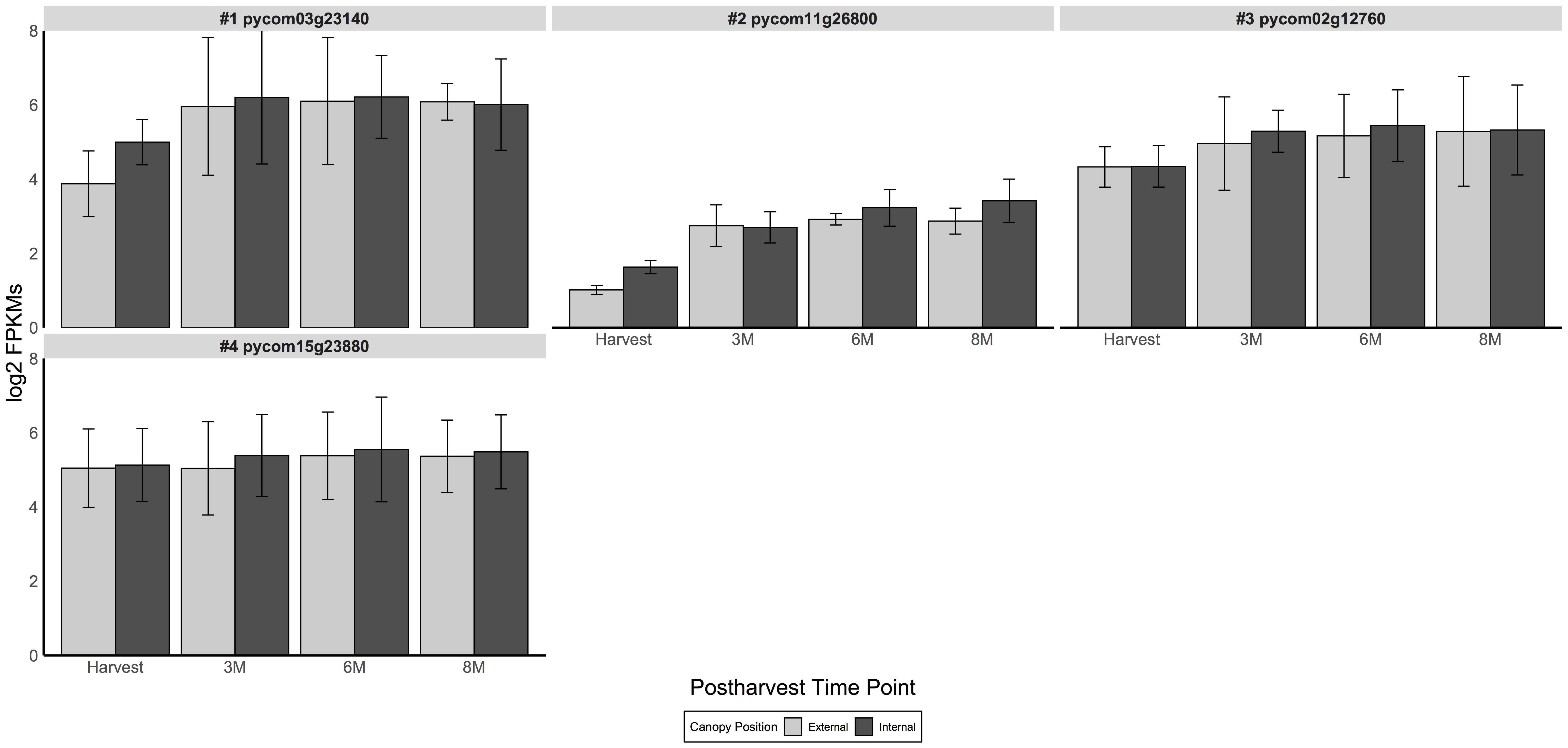

Supplement: Supplementary file 12 [file Data_Sheet_4.ZIP › Supp.4/Peel ERS2.jpg]

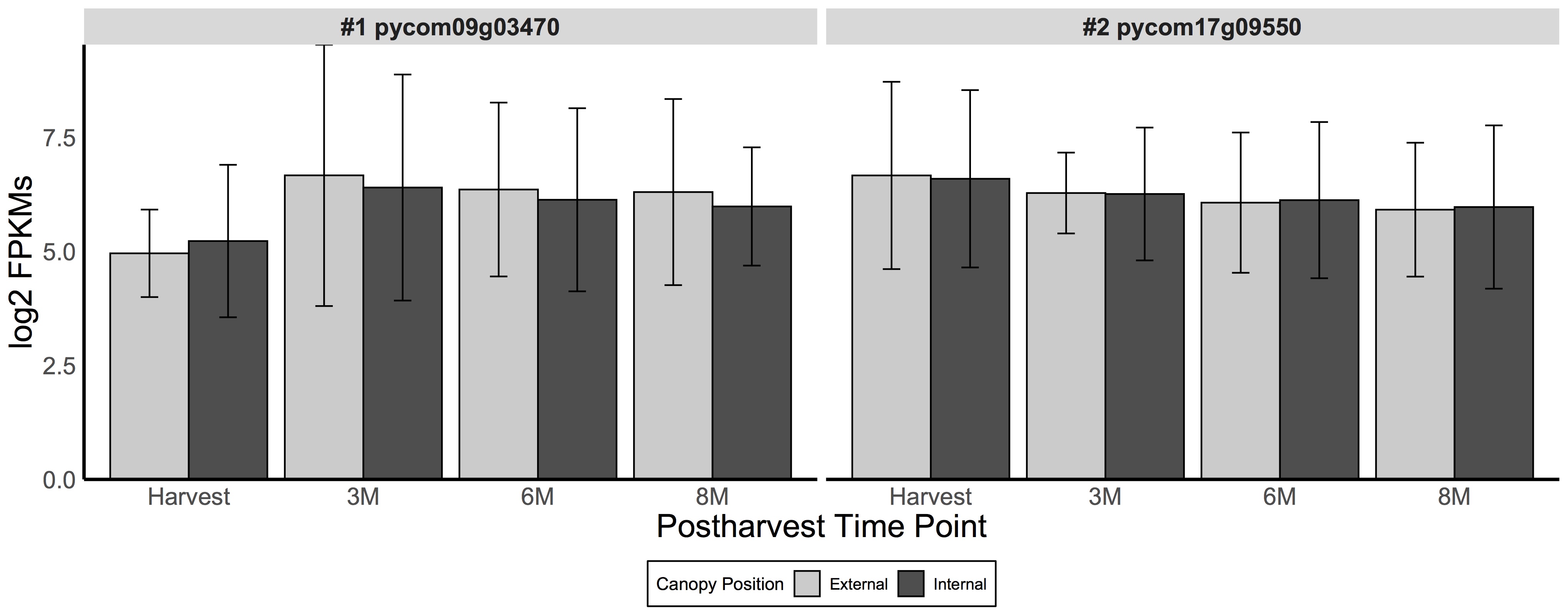

Supplement: Supplementary file 12 [file Data_Sheet_4.ZIP › Supp.4/Peel HPL2.jpg]

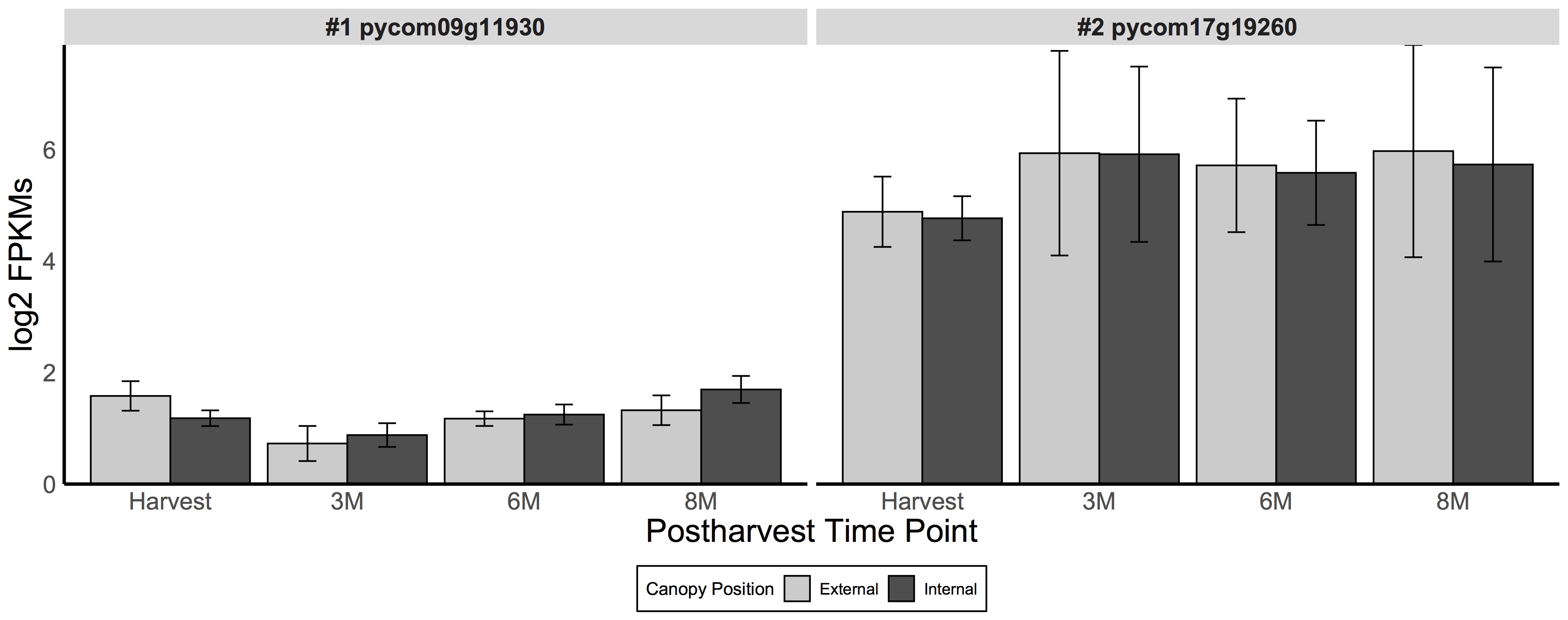

Supplement: Supplementary file 12 [file Data_Sheet_4.ZIP › Supp.4/Peel IAA Protein.jpg]

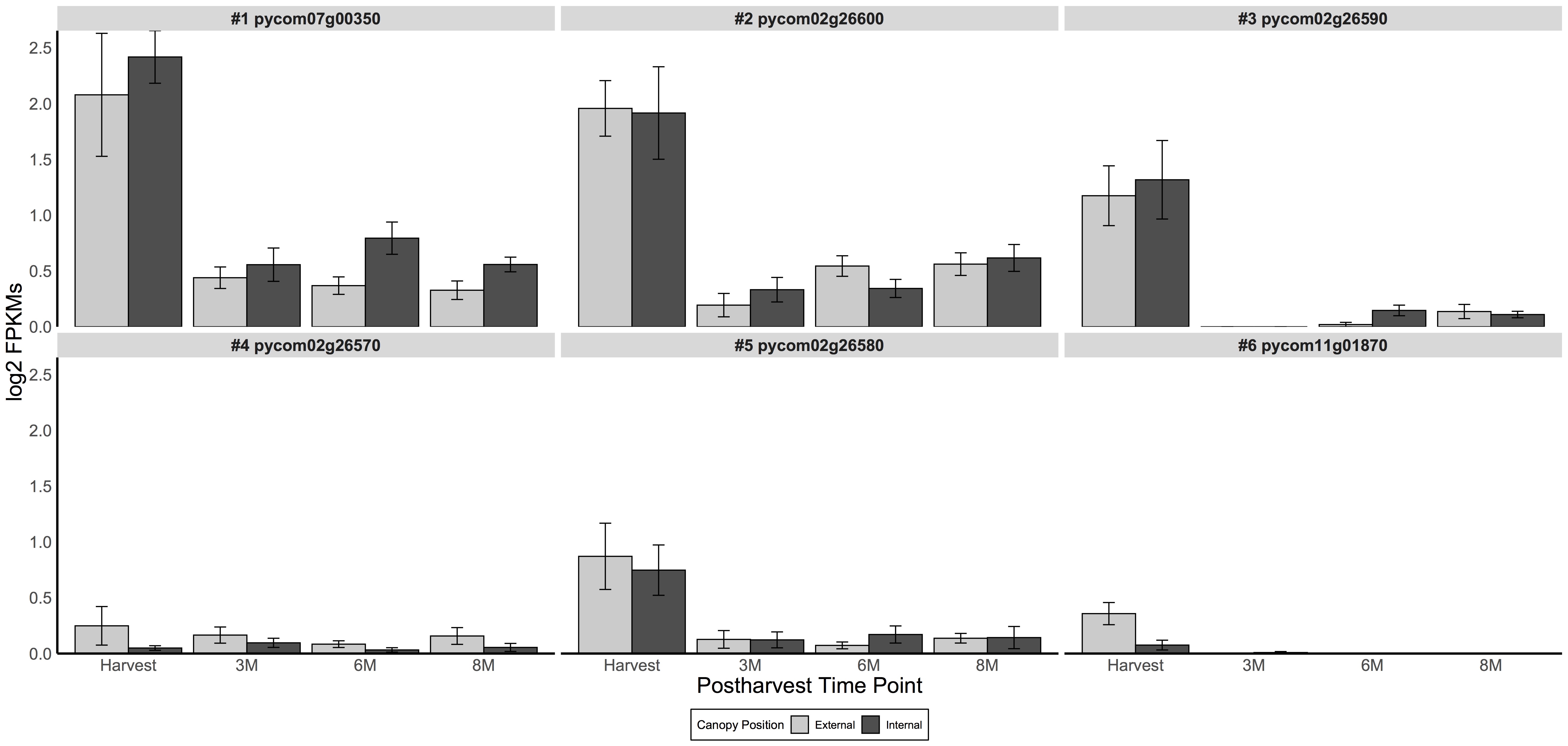

Supplement: Supplementary file 12 [file Data_Sheet_4.ZIP › Supp.4/Peel LOX.jpg]

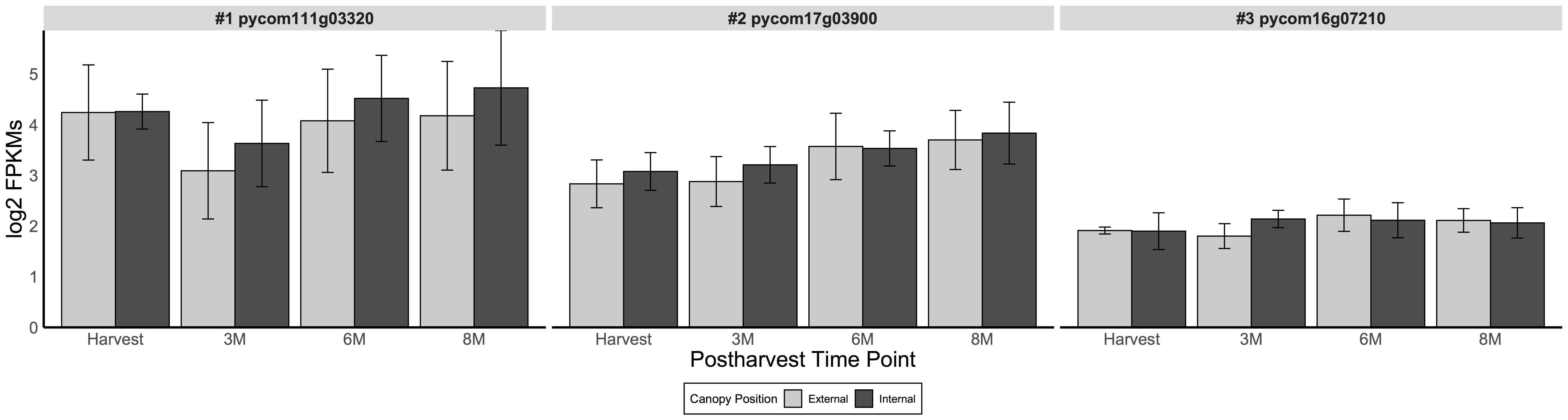

Supplement: Supplementary file 12 [file Data_Sheet_4.ZIP › Supp.4/Peel MdGene-Housekeeping.jpg]

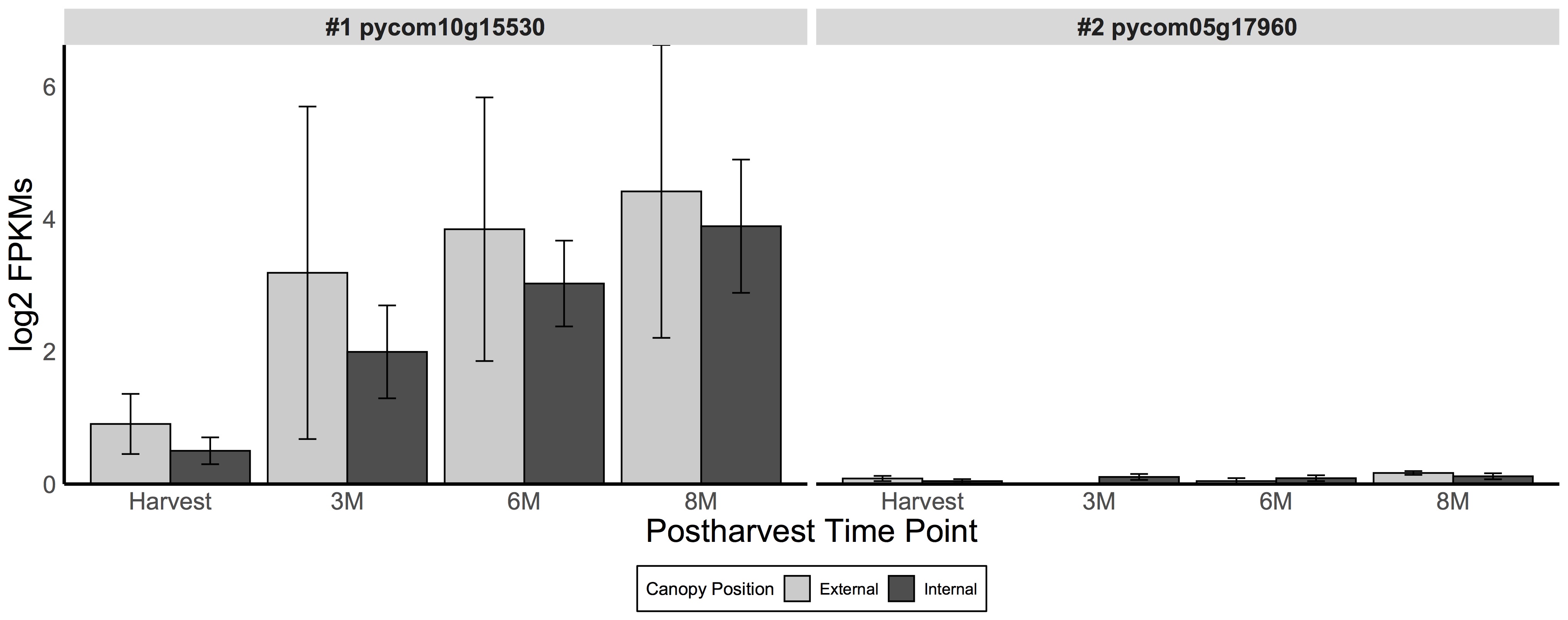

Supplement: Supplementary file 12 [file Data_Sheet_4.ZIP › Supp.4/Peel PG.jpg]
